# Independent Studies Using Deep Sequencing Resolve the Same Set of Core Bacterial Species Dominating Gut Communities of Honey Bees

**DOI:** 10.1371/journal.pone.0041250

**Published:** 2012-07-19

**Authors:** Zakee L. Sabree, Allison K. Hansen, Nancy A. Moran

**Affiliations:** Department of Ecology and Evolutionary Biology, Yale University, New Haven, Connecticut, United States of America; Uppsala University, Sweden

## Abstract

Starting in 2003, numerous studies using culture-independent methodologies to characterize the gut microbiota of honey bees have retrieved a consistent and distinctive set of eight bacterial species, based on near identity of the 16S rRNA gene sequences. A recent study [*Mattila HR, Rios D, Walker-Sperling VE, Roeselers G, Newton ILG (2012) Characterization of the active microbiotas associated with honey bees reveals healthier and broader communities when colonies are genetically diverse. PLoS ONE 7(3): e32962*], using pyrosequencing of the V1–V2 hypervariable region of the 16S rRNA gene, reported finding entirely novel bacterial species in honey bee guts, and used taxonomic assignments from these reads to predict metabolic activities based on known metabolisms of cultivable species. To better understand this discrepancy, we analyzed the Mattila et al. pyrotag dataset. In contrast to the conclusions of Mattila et al., we found that the large majority of pyrotag sequences belonged to clusters for which representative sequences were identical to sequences from previously identified core species of the bee microbiota. On average, they represent 95% of the bacteria in each worker bee in the Mattila et al. dataset, a slightly lower value than that found in other studies. Some colonies contain small proportions of other bacteria, mostly species of Enterobacteriaceae. Reanalysis of the Mattila et al. dataset also did not support a relationship between abundances of *Bifidobacterium* and of putative pathogens or a significant difference in gut communities between colonies from queens that were singly or multiply mated. Additionally, consistent with previous studies, the dataset supports the occurrence of considerable strain variation within core species, even within single colonies. The roles of these bacteria within bees, or the implications of the strain variation, are not yet clear.

## Introduction

Honey bees (*Apis mellifera*) are important in crop pollination and honey production worldwide, and have been subject to abrupt declines in recent years [1]. The causes of these losses are not yet entirely clear. One element of honey bee biology likely to be important to their health is the distinctive bacterial microbiota living in the guts of adult workers. In animals generally, more and more findings are revealing critical roles of the gut community, including protection against infectious diseases and enhancement of nutrition [2]–[4]. A critical role of the honey bee gut microbiota seems especially likely because the same set of clusters, each consisting of phylogenetically close members, recur in honey bees worldwide and comprise the majority of bacteria in each adult worker, based on studies using a variety of non-culture-based methods for examining community profiles ([5]–[11]; Table 1). The dominant, recurring honey bee-associated clusters are: “Gamma-1” and “Gamma-2” (*Gammaproteobacteria*), “Beta” (*Betaproteobacteria*), “Alpha-1” and “Alpha-2” (*Alphaproteobacteria*), “Bifido” (*Actinobacteria*) and “Firm-4” and “Firm-5” (*Firmicutes*) [9]. Full-length 16S rRNA sequences that fall within these clusters form tight clades mostly showing >97% sequence identity; hereafter, for simplicity, we refer to these as “species” or “species groups”, although they may contain multiple closely related species or “strains.” Of the eight species groups that dominate honey bee guts, five have been found only in *Apis* (honey bees and close relatives), and two are exclusively found in *Apis* species and in the related genus *Bombus* (bumblebees) [6], [9], [12]. Recent experiments on bumblebees have provided preliminary evidence that one or more of these bacterial species can prevent infection by protozoan parasites [12], raising the possibility that similar protective functions might occur in honey bees.

**Table 1:** Common honey bee gut bacterial species groups are ubiquitous and detected by multiple approaches.

Study	Locality	Sample	Method	Total *n* sequences	% known bee species groups#	Alpha 1	Alpha 2	Beta	Gamma 1	Gamma 2	Firm 4	Firm 5	Bifido	other bacteria
Jeyaprakash et al. 2003	South Africa	dissected guts	Sanger	8	n/a	+ (3)	+ (1)	+ (2)	+ (2)	-	-	+ (1)	+ (1)	*
Mohr and Tebbe 2006	Germany	dissected guts	Sanger	13	n/a	-	+ (1)	+ (1)	+ (2)	-	-	-	-	*
Olafsson and Vasquez 2008^	Sweden	isolates from guts	Sanger	17	n/a	-	-	-	+ (3)	+ (1)	+ (1)	+ (4)	+ (5)	*
Vasquez and Olofsson 2009^	Arizona	isolates from guts		11	n/a	-	-	-	+ (1)	-	+ (1)	+ (2)	+ (4)	-
Babiendrier et al. 2006	Switzerland	midgut and hindgut	Sanger	27	n/a	+ (3)	+ (2)	+ (6)	+ (8)	+ (1)	+ (2)	+ (4)	-	*
Disayathanoowat et al. 2012	Thailand	midguts	Sanger	17	n/a	-	-	+	+ (1)	-	-	+ (2)	+ (1)	*
Cox Foster et al. 2007	Australia, USA, Hawaii	pooled whole bees	Sanger	428	97.4	1.9	3.2	16.9	60.9	9.6	0.6	2.8	1.7	2.6
Martinson et al. 2011	Arizona	single whole bee	Sanger	271	98.5	0.0	1.1	11.1	11.8	0.0	10.0	63.8	0.7	1.5
Martinson et al. 2011	Arizona	bacterial cells isolated from pooled guts	Sanger	267	98.5	0.7	0.0	3.7	9.7	0.0	10.5	60.7	13.1	1.5
Martinson et al. 2012	Arizona	dissected gut sections	pyrotags 454	96,505	99.9	0.0	0.3	20.3	10.1	24.2	0.2	44.0	0.8	0.1
Mattila et al. 2012 (reanalysis)	Massachusetts	dissected guts	pyrotags 454	106,344	94.8	0.0	0.0$	6.74	49.10	1.12	11.05	21.36	5.41	5.2&
Moran et al. 2012	Arizona & Maryland	dissected guts	pyrotags 454	329,550	99.1	1.0	1.0	9.1	11.9	2.0	45.4	23.2	5.4	0.9

Honey bee species group designations after Babendreier et al. [8].

# -For studies using T-RFLP or similar method, presence is shown as “+.” Number of unique associated sequences are indicated in parentheses. For studies with random sequencing, percent representation is given.

* -Several other distant lineages retrieved but frequencies cannot be estimated due to the methods used in these studies.

^ -Sequences were mostly from cultured isolates and thus may not represent all bacteria in the gut.

$ -Reads that clustered within the Alpha-2 species group did not pass our initial filtering criteria. These reads were identified after selecting for those between 310–350 bp long. The average abundance of Alpha-2 reads in our analysis of the Mattila et al. (2012) dataset was 1.22%, SD = 0.80%.

& -Includes the CFB-1 bee species group.

Characterization of the honey bee microbiota has been attempted using a number of methods. Earlier culture-based methods (e.g., [13]) did not retrieve the most abundant members, or were not checked using modern molecular diagnostics, so those studies likely focused on organisms that could grow well under standard culture conditions and not necessarily the dominant organisms living in the gut. Non-culture based methods have been used since 2003, and have repeatedly reported 16S rRNA gene sequences representing the same eight species, although studies have used different methods and sampling depths (Table 1). For studies prior to 2011, 16S rDNA sequences were amplified, cloned and sequenced using traditional Sanger sequencing, with the largest two studies including several hundred Sanger sequences each [7], [9].

In 2012, three studies have reported deeper sampling of microbial communities through pyrosequencing of 16S rRNA gene amplicons prepared from nucleic acids extracted from honey bee guts. Two of these, [10], [14], report the same eight species in multiple samples from Arizona and Maryland and find that their proportions differ among individual bees but that together these few species comprise over 98% of the 16S rDNA amplicons from the bee guts.

A recent study by Mattila et al. [15] used a similar pyrosequencing approach to survey the active proportion of the honey bee gut microbiome by sequencing 16S rRNA gene amplicons prepared from total RNA-based cDNA libraries. Mattila et al. [15] reports predominance of completely new bacterial species in the honey bee gut microbiota and assigns these to a number of groups not reported by any other study from bees, including *Oenococcus*, *Succinivibrionaceae*, and others. To further understand this striking discrepancy, we have reanalyzed the gut-associated sequences from the Mattila et al. [15] study. We then compare results from our reanalysis to other studies and test two of their primary results using our analyses of their data.

## Methods

A total of 217,541 raw, barcoded amplicons of the V1–V2 region of the 16S rRNA gene (“pyrotags”), generated by Roche 454 FLX Titanium sequencing as described by Mattila et al. [15], were downloaded from the NCBI Sequence Read Archive (accession #DRA000526), trimmed to remove pyrosequencing adaptors, low quality base calls (<27 Phred score) and size-selected (≥350 bp) within CLC Workstation (www.clcbio.com). When downloading the primer, barcode and adapter sequences from the Sequence Read Archive website, we noticed that the forward primer sequence deposited in the Archive had a “G” at the 12^th^ position substituted for an “M,” which is the IUPAC code for an “A/C” degeneracy, as described in the Lane et al. [16] reference cited in Mattila et al. [15]. Use of the forward primer with the “G” substitution in the raw read preprocessing, which includes a quality filtering step that excludes reads with errors in the primer sequence, resulted in loss of all reads. We changed the “G” in the primer sequence to an “M” and were able to proceed with nearly all of the reads to subsequent quality-filtering steps and sequence similarity clustering. A web-implementation of Pyrotagger [17] was used to select for reads whose barcode and forward primer sequences were 100% error-free, had a Phred score of ≥27 over ≥90% of the sequence, were between 350–400 bp long and were not flagged as possible chimeras. High-quality sequence reads were clustered based on ≥97% sequence identity via uclust [18] into operational taxonomic units (OTUs). Sequences representative of each OTU were taxonomically classified by blastn-based comparison to greengenes and silva databases within Pyrotagger as well as to a local NCBI non-redundant ‘nt’ nucleotide sequence database ([19], downloaded June 29, 2011).

To remove noise from the data, including potential rare contaminants, we removed OTUs that did not meet the criterion of being present as at least two sequences in each of at least two samples; this resulted in elimination of only 0.14% of the reads. The resulting set of OTUs was used in diversity analyses (see below).

To assign taxonomic designations to OTUs, we used representative sequences, selected as the most frequent sequence within each OTU, in blastn searches against the NCBI non-redundant ‘nt’ nucleotide sequence database (March 29 2012). For classifying OTUs with top hits (at 99–100% identity) to a representative of one of the previously retrieved species from bees, we used the designations first applied by Babendreier et al. [8], and subsequently used by Cox-Foster et al. [7], Martinson et al. [9], [10] and Moran et al. [14]. These classifications were applied if the match in blastn was 99–100% to a bee-associated bacterium and if blastn gave no other close matches. We then designated the species by examining the associated GenBank file of top matches. In the case of the previously studied bee-associated bacterial species, this designation is indicated in a “note” field for sequences from Martinson et al. [9], [10] and is given in the publication for sequences from Babendreier et al. [8].

In some cases, different OTUs had top matches, at 100% identity, to sequences assigned to the same species, due to the presence of several slightly different 16S rRNA sequences for a species in the database. In these cases, we grouped the OTUs into the same species, to facilitate visualization of the relative abundances of species present in each sample. We refer to these single or clustered OTU groups as species. (Previous studies have sometimes referred to these as ‘phylotypes’, in recognition that each may include distinct strains with different ecologies and gene sets). We note that sequences with <97% identity for the amplicon containing hypervariable V1–V2 regions (corresponding to these pyrotags) sometimes have >97% identity for the overall 16S rRNA, with the result that different OTUs may have top hits to sequences that would be considered the same species under a 97% criterion for species delineation. Also, we note that the 97% OTUs generated directly by clustering were used in analyses of diversity, as described below.

In order to clarify apparent discrepancies between conclusions of Mattila et al. [15] and other findings for the honey bee gut community composition, we obtained representative sequences for the main taxonomic assignments as reported in Table 1 of Mattila et al. [15] from the corresponding author (I. Newton) of the study. We used these sequences in web-based implementations of the Ribosomal Database Classifier and SeqMatch [20] (RDP database, release 10, update 28), and in blastn searches against the NCBI nucleotide database (searches performed on March 27, 2012). We recorded the SeqMatch classifications and also the top hits in the NCBI database, including the percent nucleotide identities, and compared these to the assignments and percent identities for the taxonomy assignments reported in Table 1 of Mattila et al. [15]. We designated species using similar criteria as used for representative sequences of our OTUs.

Our quality-filtering criteria yielded almost twice as many sequences from the Matilla et al. [15] dataset, but fewer OTUs. We reanalyzed the gut-derived reads, using criteria for quality and clustering similar to those described by Mattila et al., using the Mothur platform [21] with requirement of quality score >27 for 97% of the read, and length between 300 and 400 bp. OTUs were at 97% identity. Rare OTUs were excluded using the same rule as above.

We retested other conclusions of Mattila et al. [15] regarding bacterial community patterns. We used our clusters, generated as described above, for these tests. We restricted our analyses to the bee gut communities, because communities in bee bread are distinct from those in guts and cannot be pooled in the same analyses. In our analyses we used the samples designated by “bgmdi” and bgsdi”, and we simplify these tags in this paper, using “M” and “S” for colonies with multiply and singly mated queens respectively, followed by a number designating the colony sampled. Colonies with multiply and singly mated queens are presumed to differ in genetic diversity among workers.

First, we tested a potential relationship between number of times the queen mated (singly versus multiply) and the gut bacterial communities, using several approaches. To determine whether colony genetic diversity affected the abundances of putative pathogens, we performed a Mann-Whitney U test on the ratio of putative pathogen numbers divided by total sequences per sample. The putative pathogen category included sequences from the *Enterobacteraceae* and from *M. plutonius*, similar to the pathogen category as defined in Mattila et al. [15] (We agree with Mattila at al. [15] that these *Enterobacteriaceae* might speculatively be considered to be pathogens, mostly based on their erratic distributions among individual bees and colonies).

Second, we tested whether gut community composition or diversity differed between colonies with singly and multiply mated queens. For all community multivariate analyses, PC-ORD (version 4.25) was used [22]. Analyses were conducted on the 42 OTUs that resulted from clustering the data, with elimination of extremely rare OTUs, as described below. Bacterial sequence reads per colony were standardized to the same sample size before diversity and multivariate community analyses were conducted. Standardization was carried out by randomly selecting 1230 reads (the smallest sample size) per colony using a custom Perl script. For nonparametric community structure and composition analyses, Multi-Response Permutation Procedures (MRPP) [23] were used to test for differences among *a priori* groups (singly and multiply mated queen colonies). Nonmetric Multidimensional Scaling (NMS) [24]–[25] was used to visualize differences among honey bee gut microbiotas between bee colonies from both groups. Outlier Analysis [22] identified bee colonies S5 and S7 as extreme outliers, and they were removed because such outliers can distort ordination solutions. All criteria, distance measures, and parameters chosen are similar to those in Hansen et al. [26]. Briefly, a three-dimensional solution was chosen based on a combination of low stress, final instability, and P-values; 500 iterations were conducted. For ease of visualization, the two axes explaining the highest amount of variation in the dataset were presented in a 2-D figure. For alpha diversity analyses, colonies were considered subsamples of each treatment (N = 10 and 12, respectively for singly and multiply mated queen colonies). Species accumulation curves were plotted with error bars (plus or minus two standard deviations) to contrast species diversity between the two treatments, and to evaluate the adequacy of dataset sample size. Subsampling was repeated 500 times for this analysis and the number of OTUs and average distance (Sorensen) for each subsample size was computed. Using EstimateS [27] evenness of bacterial OTUs for each treatment was estimated using Simpson's dominance index (D) [28], and richness for each treatment was calculated using the Chao 2 richness index [29].

The above diversity analyses were repeated for the alternative OTU set, based on 166 retained OTUs.

Finally, we performed a Pearson's correlation analyses to test the proposed relationship between the number of *Bifidobacterium* cells and numbers of putative pathogens in the gut microbiota. We analyzed gut microbiota samples only for a relationship between numbers of sequences corresponding to *Bifidobacterium* and numbers of sequences corresponding to putative pathogens as defined above.

## Results

### Number of reads analyzed and OTUs generated

After filtering we retained 106,344 total sequences or 48.9% of the raw reads (Table 2). This was about twice the 56,556 reads from guts used by Mattila et al. The difference reflects a larger number of raw reads initially retrieved prior to filtering; we used lower quality score criteria but a higher length cutoff.

**Table 2:** Mattila et al. (2012) dataset pyrotag processing summary.

Raw reads%	217541
Reads passing quality control criteria^	106497
Average read length	350 bp
Initial OTUs	251
Final OTUs, post-frequency filtering&	42 (99.86%)
Final reads	106344
Average *n* sequence reads per sample	4,833 (range: 1,230–9,934)

% -Downloaded on March 25, 2012 from the Sequence Read Archive (SRA accession number DRA000526).

^ -Raw sequence reads were trimmed to remove low quality (<27 Phred score) base calls and reads that had a) 1 or more errors in the barcode or primer regions, b) a <27 Phred score in >10% of the sequence, or c) were <350 or >400 bp long were omitted.

& -OTUs not represented by at least two sequence reads in at least two samples were omitted from final OTUs that were used in our analysis. Proportion of total, high-quality reads after frequency-filtering shown in parentheses.

We obtained 251 OTUs, with 97% identity cutoffs, for the bee gut samples from Mattila et al. [15] (Table 2). Of these OTUs, 209 did not meet the criterion of being present as at least 2 sequences in each of at least 2 samples. These 209 clusters together comprised only 0.14% of the total sequences. The remaining 42 OTUs captured 99.86% of all reads, indicating that most sequences fell within a few large OTUs. These 42 OTUs were used in diversity analyses (see below).

### Taxonomic classification of OTUs

**Figure 1:**
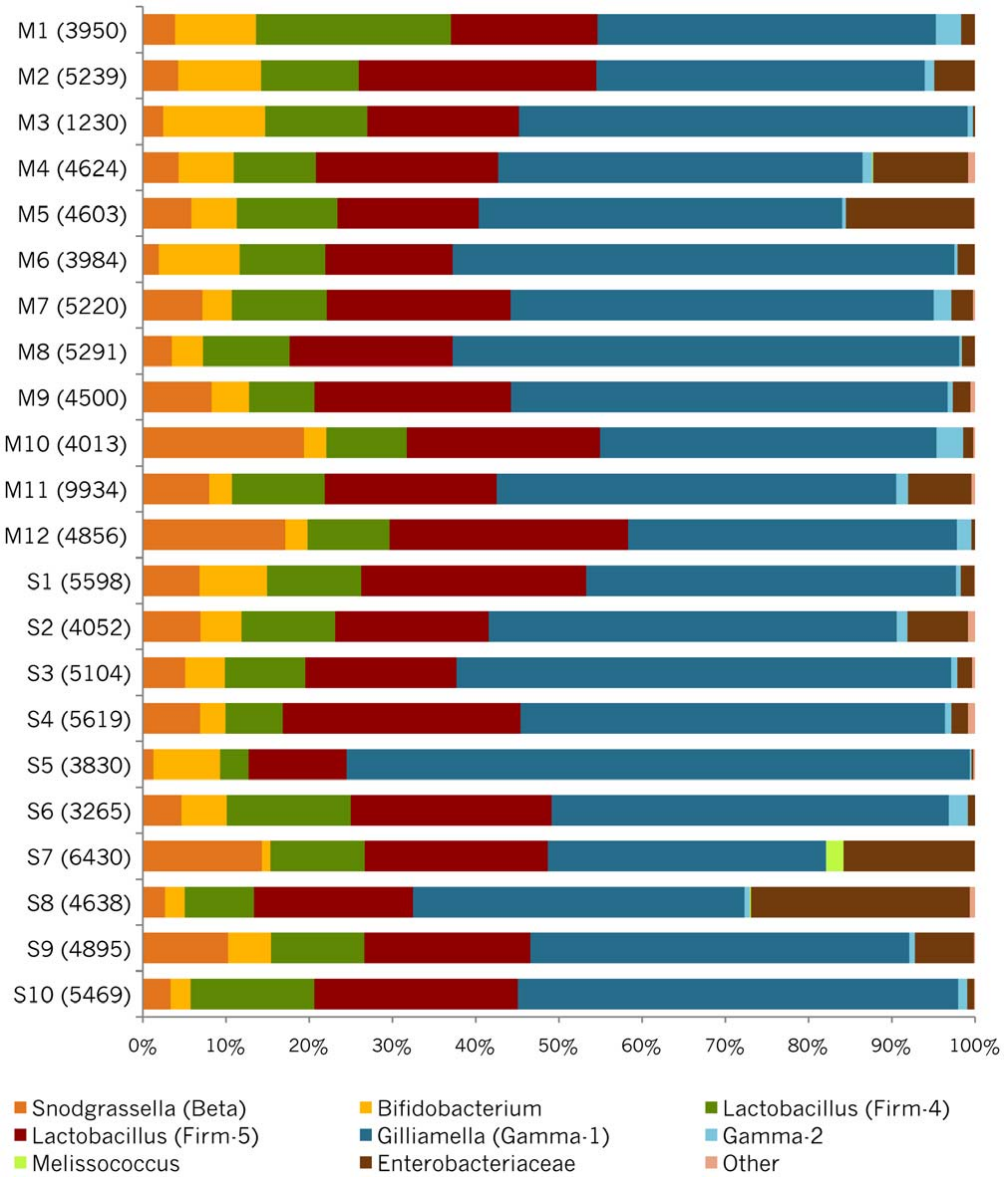
Relative abundances of the typical bee-associated bacterial species in the Mattila et al. (2012) dataset. “Other” includes the CFB-1 species group, which was uncommon in all samples.

Taxonomic assignments were based on the top blastn hits of representative sequences of each OTU (listed in Text S1). In most cases, sequence identity was 100% to sequences of characterized species. These species included previously recognized species dominating the gut communities of honey bees, and we use established labels [7]–[10], [14], [30] to refer to these species. Of the 42 OTUs, 23 corresponded to distinct species (i. e., 1 OTU = 1 species). The other 19 OTUs were grouped into a total of six species, all previously associated with bee samples: ‘*Gilliamella apicola* (Gamma-1),’ ‘Gamma-2’, ‘*Snodgrassella alvi* (Beta)’, ‘Firm-4’ (*Lactobacillus* sp.), ‘Firm-5’ (Lactobacillus sp), and ‘Bifido’ (*Bifidobacterium* sp). Representative sequences for these bee-associated OTUs were identical to published sequences for these species [5], [7]–[8], [10]–[11]. Together, these comprised an average of 94.79% (SD = 6.75%) of all sequence reads per sample, and each of these six species groups was present in every sample (Fig. 1). An additional species, represented by a single OTU and averaging 0.15% per sample, was represented by a sequence with 99% identity to an uncultured member of the *Bacteroidetes*, ‘CFB-1’, identified in honey bee guts by Babendreier et al. [8].

Two of the species commonly found in bee guts, Alpha-1 and Alpha-2, were not included among our OTUs. This was puzzling because the Alpha-2 full-length 16S rRNA was previously shown to have near-identity to the sequence from *Saccharibacter floricola* [9], and Mattila et al. [15] state that “*Saccharibacter*” was present in their honey bee gut samples. In order to determine the basis of this discrepancy, we modified our read length selection criteria to include reads 310–350 bp long (as in the Mattila et al. analysis) and retrieved reads that formed a cluster with a representative sequence showing 100% identity to a sequence within the Alpha-2 group (GenBank accession #AY370188), as defined by Babendreier et al. [8] and found in most other studies of bee gut microbiota (Table 1). These Alpha-2 sequences were present in all of the gut samples at an average of 1.3% per sample. Likewise, specific searches for Alpha-1 revealed its presence as perfect matches in four samples (M1, M5, S2 and S9); it was not included in our retained OTUs, because it was represented by single sequences in three of the four samples, and thus did not meet the criterion of at least two sequences in at least two samples.

Of an additional 19 OTUs, 18 had best blast hits, mostly at 100% identity, to distinct species of *Enterobacteriaceae* detected in various environmental samples including other insect guts, wastewater treatment effluent, mammalian guts, plant surfaces and soil. These sequences comprised an average of 4.95% (SD = 6.85%) of all reads per sample, and many of these small OTUs were erratically present across samples (Table S1). Another OTU had a 99.1% identity to the causative agent of European foulbrood, *Melissococcus plutonius* (NCBI accession #AJ301842). This *M. plutonius* OTU was infrequently detected in the singly mated (2/10) and multiply mated (2/12) honey bee gut samples, and in only one singly mated colony (S7) did it comprise >1% of the total sequences in the bee guts. Our analysis shows that the same bacteria that have been previously observed in honey bee guts largely dominate the gut microbiomes for both colony types.

To verify that we were examining the same sequences corresponding to the groups discussed in Mattila et al. [15], we analyzed sequences sent by the corresponding author as representatives of the taxon assignments listed in Table 1 of their paper and discussed in their Abstract, Results, and Discussion. Results of our analyses, and comparisons with their designations, are reported in Table 3. Of the 15 OTUs listed in their table, 11 corresponded to the species previously found to dominate honey bee gut communities, and four represent species of *Enterobacteriaceae*.

**Table 3:** Many of the most active taxa from the Mattila et al. [15] study are identical to known bee species groups.

Taxonomy assignment in Mattila et al (Table 1)	Sequence tag in Mattila et al. dataset	Highest identity to Mattila et al taxon	Species* assignment	Highest identity of hit to known bee species groups	Representative accessions for bee species group hits
Succinivibrionaceae	GPEDTIY04JG0L5	85%	Gamma-1 = Gilliamella	100.0%	HM111884
Unclassified	GPEDTIY04IRBP7		Gamma-1 = Gilliamella	100.0%	AY370191
Bowmanella	GPEDTIY04ITH5U	87%	Gamma-1 = Gilliamella	100.0%	DQ837611
Saccharibacter	GPEDTIY04H0LN3	91%	Alpha-2	100.0%	AY370188
Laribacter	GPEDTIY04IQ7R3	92%	Beta = Snodgrassella	99.7%	DQ837621
Shimazuella	GPEDTIY04IHRSU	81%	Firm-5	100.0%	DQ837636
Oenococcus	GPEDTIY04H54P4	79%	Firm-5	100.0%	HM111883
Rummeliibacillus	GPEDTIY04IHMXD	85%	Firm-5	100.0%	HM111947
Paralactobacillus	GPEDTIY04IKZSR	84%	Firm-4	100.0%	HM111967
Atopobacter	GPEDTIY04JDBJG	81%	Firm-4	100.0%	HM113352
Bifidobacterium	GPEDTIY04I6NP3	100%	Bifido	100.0%	HM113215
Enterobacter	GPEDTIY04I75AG	100.0%	Enterobacter (Pantoea, Morganella/Klebsiella oxytoca)	100.0%	
Brenneria	GPEDTIY04JNK65	100.0%	Brenneria/ant lion symbiont	100.0%	
Klebsiella	GPEDTIY04IJBJS	100.0%	Klebsiella	100.0%	
Escherichia/Shigella	GX4R3OO03FL1O6	100.0%	Escherichia/Shigella	100.0%	

* -Species tags for known bee-associated species are the same as those in [7]–[10] and [14].

For these 11 representative sequences, nucleotide identities to the closest representatives of the previously retrieved species were 100%. Nucleotide identities to the taxa designated in Mattila et al. [15] were far lower, ranging from 79 to 92% (Table 3). In that paper, completely different taxonomy assignments were given even when representative sequences fell within the same tight clusters that we refer to as species/species groups. For example, for *Gilliamella* (Gamma-1), two representative sequences were classified differently, although each had a 100% match to previously sequenced representatives of this species (reflecting some of the strain diversity within this species). These representative sequences from Mattila et al. had pairwise divergence of 1.2%, versus 0.9% for the full-length sequences to which they had perfect matches, but were classified as *Succinivibrionaceae* and *Bowmanella* despite being 13% and 15% divergent from the closest representatives in those named taxa. For the Firm-4 and Firm-5 sequences, divergences from the taxonomies assigned in Mattila et al. ranged from 15–21%. For the three sequences with 100% matches to the Firm-5 species group, pairwise divergences were 3.7–5.2% for the pyrotag sequences and 1.8–3.1% for the full-length 16S rRNA sequences to which the pyrotag reads had perfect matches. For the two representative sequences from Mattila et al. that we found to have perfect matches to sequences in the Firm-4 species, the divergence was only 0.6%, versus 0.3% for the full-length sequences, yet were classified as two distinct genera (*Paralactobacillus* and *Atopobacter*) in Mattila et al. Thus, the Mattila et al. [15] sequences capture some of the strain variation previously found within the typical species of honey bee guts, but tend to show higher variation due at least in part to inclusion of the V1–V2 hypervariable region. For the full-length 16S rRNA, divergence within these species (or species groups) is usually <3%, but the fragment represented by these data (containing the V1–V2 hypervariable regions) is more divergent than the average for the gene.

The other four representative sequences listed in Table 1 of Mattila et al. [15] fell within *Enterobacteriaceae* and showed 100% identities to species within the genera *Enterobacter*, *Brenneria*, *Klebsiella*, and *Escherichia*, the same taxa reported by Mattila et al. These also correspond to some of the OTUs recovered in our clustering, and received the same species designations in our clustering.

Thus, the Mattila et al. [15] dataset for guts was dominated by the same species found to dominate in honey bee guts in numerous previous studies, which used a variety of methods and regions of the 16S rRNA gene (Table 1).

As noted, OTUs taxonomically assigned to Alpha-1 and Alpha-2 were not included in our community diversity analysis, but these species were detected when criteria for inclusion were relaxed. Alpha-1, was very scarce and completely absent from most colonies; this species was also absent from some samples in another pyrotag study with deep sequencing [14]. Another species, Alpha-2 (*Acetobacteraceae*), was present in all colonies and never abundant (∼1.3%), as observed in some other datasets [9]–[10], [14]. Interestingly, the Mattila et al. [15] dataset included a low abundance of reads with 100% identity to “CFB-1”, as retrieved by Babendreier et al. [8]; this organism is related to species of *Bacteroides*. These rarer organisms could be important in honey bee biology, but if their typical abundances are <10^−5^, they are difficult to assay even by deep sequencing methods.

### Genetic diversity and honey bee microbiome community diversity

A major conclusion of Mattila et al. [15] was an effect of number of queen matings on the diversity of the bacterial communities in bees within colonies. We used the 42 OTUs generated by our 97% clustering, which incorporated almost twice as many reads from bee guts as used by Mattila et al. [15] and which reflected different filtering criteria, to determine whether we could detect any effects of number of queen matings.

**Figure 2:**
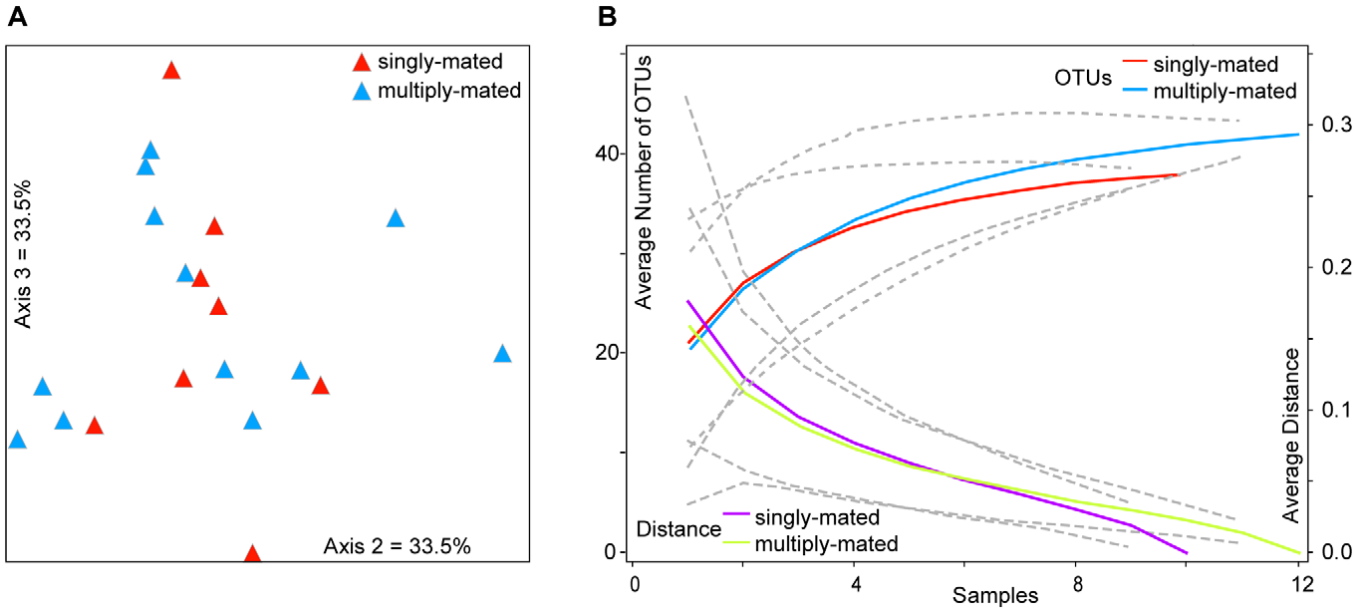
Analyses comparing microbiota between colonies with singly mated and multiply mated queens. (A) NMS ordination plot showing the two axes representing most of the variation among honey bee gut communities (N = 20). Samples S5 and S7 were omitted as outliers. (B) Species accumulation curves for the two treatments. Dotted lines indicate confidence intervals (±2 SD) for species and distance curves.

First, we performed community analyses to test whether the two colony types showed any significant differences in composition or diversity in their gut microbiotas. To determine whether assemblages differ between singly and multiply mated queen colonies, multivariate analyses (NMS and MRPP) were used. Bacterial community profiles were not significantly different between the two types of colonies (without outliers excluded, MRPP: *T* = 1.4406, *A* = −0.02032, *P* = 0.981; with outliers excluded MRPP: *T* = 1.2424, *A* = −0.01913, *P* = 0.930). NMS ordination results reflect this lack of difference as bee colonies did not cluster by colony type (Fig. 2A; 3D solution, 500 iterations, final stress = 9.333, final instability = 0.00035, Monte Carlo P = 0.0392. Variation explained- Axis 1 = 24.2%; Axis 2 = 33.5%; and Axis 3 = 33.5%).

To determine if there was a significant difference in richness between colony types, OTU accumulation curves were generated for each treatment. Based on OTU accumulation curves, the confidence interval for the multiply mated queen colonies encompasses the confidence interval and OTU curve for the singly mated queen colonies (Fig. 2B), revealing no definitive difference in OTU richness between the two groups. To measure alpha diversity, Chao 2, which is suitable for small sample sizes [31], was computed. Community richness for the multiply mated queen colonies was similar to that for singly mated queen colonies, when considering the 95% confidence intervals for these estimates; multiply mated group: 44.75 (42.45–58.68 95% CI); singly mated group 38.77 (38.08–45.39 95% CI). The other metric of diversity, evenness, was not different between colony types (Simpson's Mean = 3.80 for multiply mated and 3.73 for singly mated).

### Impact of genetic diversity and *Bifidobacterium* abundance on pathogen load

We also tested whether number of queen matings affected the abundance of putative pathogens in worker guts. As in Mattila et al. [15], we grouped the 18 *Enterobacteriaceae* and *Melissococcus* species groups as putative pathogens. The proportion of putative pathogens in the pyrotags from gut microbiotas of workers from singly mated versus multiply mated colonies did not differ significantly (Mann-Whitney U, 1 tailed test, p = 0.245).

**Figure 3:**
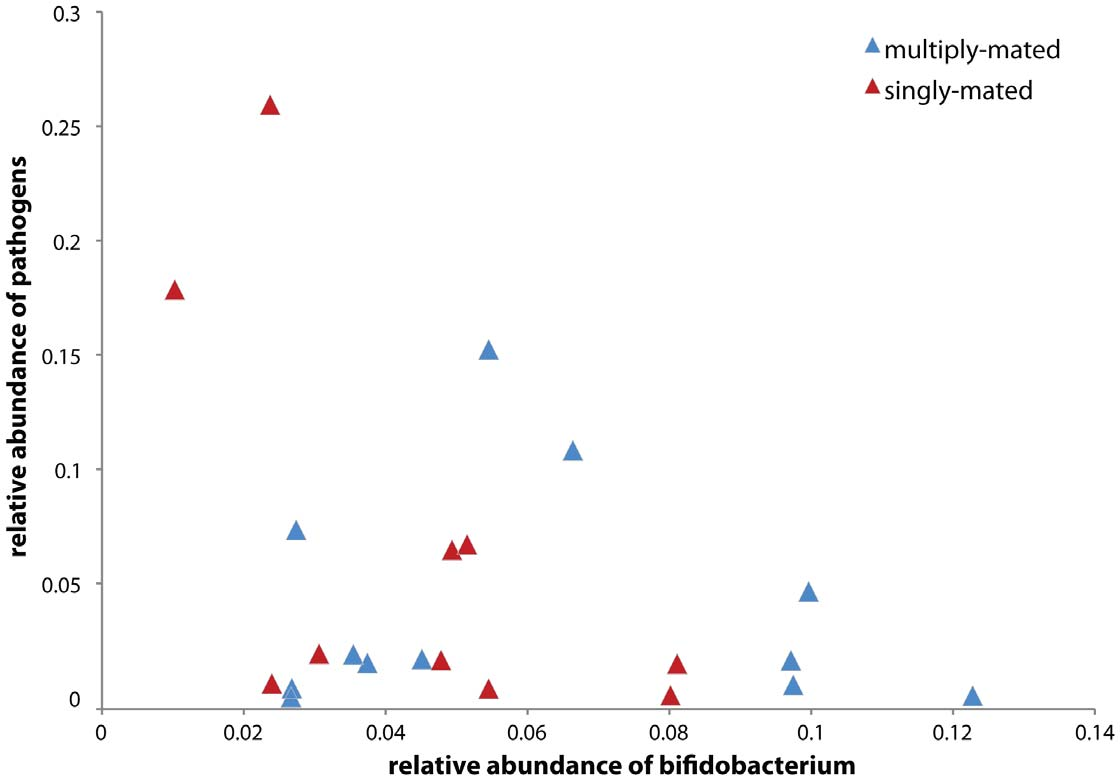
Relationship between *Bifidobacterium* and putative pathogens in the Mattila et al. (2012) dataset. Putative pathogens include *Enterobacteriaceae* and a known larval pathogen, *Melissococcus plutonius*.

Mattila et al. [15] also reported that abundance of *Bifidobacterium* was negatively correlated with level of putative pathogens, using the same definition for pathogens. Using our OTUs, we tested this proposed relationship. We retrieve a somewhat similar pattern, highly influenced by two singly mated colonies, S7 and S8, that had *M. plutonius* infections and the highest numbers of reads for the overall “pathogen” category (Fig. 3). However, we found that the relationship is not significant (Pearson's correlation, 1-tailed test, p = 0.36). The analysis reveals variation among samples in pyrotag abundances of both *Bifidobacterium* and putative pathogen species groups, but this is erratic with no clear link to whether the colonies had queens that received sperm from a single or multiple donors.

### Alternative analysis with different quality and clustering criteria

We conducted an independent analysis using lower length criterion and alternative quality score filters, and carried out clustering with the Mothur package [21] instead of UClust. Although this retained even more reads and resulted in more OTUs, general results were similar, with 95% of reads belonging to OTUs assigned to species previously retrieved from bees. Analysis of 166 retained OTUs again did not reveal any significant differences between the gut communities in colonies with singly and multiply mated queens. Full description of these results is given in Text S2.

## Discussion

Mattila et al. [15] claimed to find novel groups of bacteria in honey bees. For example, they state “Importantly, the two dominant genera identified by our study (*Succinivibrio* and *Oenococcus*) have never been identified in honey bee colonies, which suggests that the major microbial players that are associated with honey bees were overlooked by previous methodologies.” In fact, 95% of the sequences they obtain from guts correspond to bacteria previously identified from honey bee guts, and their representative sequences that they assign to taxa such as “*Succinivibrio*” and “*Oenococcus*” show 100% identity to sequences from previous studies in honey bees (Table 3). The basis for their conclusions is not clear. Possibly only cultured isolates were considered in their analyses. We note that they report using a 60% confidence cutoff for assigning taxonomy descriptions. This criterion is expected to lead to some incorrect assignments, especially in combination with elimination of sequences from non-culture based studies.

Although earlier studies using Sanger sequencing produced fewer sequences, most of these relied on methods such as terminal restriction fragment length polymorphism to enable detection of rarer sequences (e.g. [5]–[6], [8]). Such methods enabled successful retrieval of the more common species with limited numbers of sequences (Table 1). Other studies based on Sanger sequencing did recover hundreds of sequences per sample [7], [9] and thus gave reasonably complete pictures of the diversity in the bee gut communities. The Mattila et al. [15] dataset provides a further confirmation that six of the eight species are essentially universal in honey bees, having been retrieved in all studies with more than a few dozen sequences. Previous Sanger studies used a variety of different conserved primer sites, often for the near full-length 16S rRNA gene; some used restriction fragment methods for selecting distinct, lower frequency sequences within samples. Thus a broad variety of primers and methods have produced sequences and signals for the same species. The three studies using pyrotags and deep sequencing show that these are the vast majority of organisms living in honey bee guts.

Two of these species recently have been given *Candidatus* names: “*Candidatus* Gilliamella apicola” (formerly called Gamma-1) and “*Candidatus* Snodgrassella alvi” (formerly called Beta) [10]. In honey bees, these two organisms form distinctive biofilm-like aggregations on the lining of a small region of the hindgut called the ileum [10]. Strains corresponding to *Gilliamella* and *Snodgrassella* also have been found in bumblebees in which they have been linked to protection against protozoan parasites [12]. Another pyrotag-based study showed that these two species were present in every individual of 40 bees sampled in Arizona and Maryland [14]. Furthermore, that study documented extensive strain variation within both *Gilliamella* and *Snodgrassella*, as well as recombination among 16S rRNA sequences within these species groups. This strain variation is also apparent in a metagenomic sequence analysis based on full genome sequences from honey bee guts [30]. Also present in every individual were Firm-4 and Firm-5 species, representing distinct clades within *Lactobacillus*, and a *Bifidobacterium* species group, most closely related to *Bifidobacterium* retrieved previously from bees [5], [32].

The species designated as “Gamma-2”, which is related to *Gilliamella*, but which forms a distinct clade, was recovered from every individual or colony sample in every study. The larger clade containing both Gamma-2 and *Gilliamella* also contains other sequences associated with insects [10], [33] as well as a single cultured isolate that was recently given official nomenclature [34]. This deeper clade of *Gammaproteobacteria* forms a lineage that branches near the divergence of the *Enterobacteriaceae* and the *Pasteurellaceae* but that is distinct from either [9]–[10], [33]–[34]. Some of the sequences within this clade have incorrect taxonomic information within the GenBank ‘definition’ and/or ‘organism’ fields, as labels were sometimes given on the basis of the best blastn hit using current databases.

Another distinctive aspect of the Mattila et al. [15] study was the use of RNA rather than DNA as starting material, an approach that is expected to more reliably represent only living cells. Whereas most studies on gut microbiota have sampled DNA and amplified 16S rDNA using conserved primers, Mattila et al. [15] sampled RNA and used conserved primer sites for PCR amplification from cDNA. Also, their study targeted the V1–V2 region of the 16S rRNA, whereas the two other pyrotag studies targeted the less variable V6–V8 region [10], [14]. These differences may explain why the Mattila et al. [15] dataset contained a somewhat larger number of OTUs compared to the other two pyrotag studies of bee guts. We retained 42 OTUs from their dataset, after eliminating OTUs with extremely few sequences, mostly singletons. These singletons or very small clusters can represent sequencing error or contamination of individual samples [35], and together comprise only 0.14% of the reads. Of the 42 OTUs remaining, eight correspond to the *Gilliamella* species group, one to Gamma-2, two to *Snodgrassella alvi*, two to Firm-4, four to Firm-5 and two to *Bifidobacterium* sp. Use of the V1–V2 regions is expected to yield about twice as many OTUs as use of the V6–V8 region [36]. Also, using cDNA introduces two additional steps at which base changes (and thus diversity) might be introduced: transcription within the cell and reverse transcription for cDNA production. The pyrotag approach does result in the generation of some erroneous sequences and clusters and makes the assessment of very rare OTUs problematic [35], Of the 42 OTUs, almost half were classified as distinct species within *Enterobacteriaceae*, and these must represent real organisms present in the samples.

Although the core honey bee gut species are similar across studies, the relative abundances of species within samples vary widely. In part, this variation results from differences in methods, including different DNA extraction methods and different PCR primers. Even when methods were identical for samples within a study, profiles showed substantial variation in the abundance of community members from individual bees and among colonies (Fig. 1) [10], [14].

The Mattila et al. [15] study contained samples for 22 colonies, and these differed in relative abundances of species (Fig. 1). A major conclusion of that study was that colonies with singly mated queens had less diverse microbiotas than colonies with multiply mated queens, and that greater diversity was “healthier”. In fact, Mattila et al. also found that most standard measures of diversity showed extensive overlap between the colony types (Table S2 of Mattila et al.), and a test of differences between colony types for a standard diversity measure did not support differences, in agreement with our analysis. Their evidence for differences in diversity was based on a bootstrap resampling simulation for which the 95% confidence interval for the difference in species diversity between paired samples representing the colony types did not overlap zero. We used more data, applied a more stringent length filter, and eliminated the 0.14% of reads falling in extremely rare OTUs.

We found no support for a difference between the colony types in our reanalysis of their data, nor did we find a relationship between *Bifidobacterium* and abundance of putative pathogens. We note that no data are available that show a relationship of microbiota diversity to health of colonies or individual bees. Indeed, if some of the microbial diversity consists of pathogens, then greater diversity might be as likely to represent disease as health.

Mattila et al. [15] suggest that the sequences corresponding to species within *Enterobacteriaceae* represent pathogenic organisms. Assignment of ecological roles or metabolic capabilities on the basis of a short sequence is at best tentative. For example, the sequence assigned to *Brenneria quercina* (a plant pathogen) is also identical to sequences retrieved from a mutualistic symbiont of antlions [37], and, in general, a large number of insect mutualists and commensals are found within the *Enterobacteriaceae*. Even a full-length 16S rRNA sequence gives essentially no information regarding whether the organism is beneficial or harmful to hosts, in the absence of work on the particular association. In the case of the honey bee gut microbiota, members of the *Enterobacteriaceae* species groups are erratically present among individuals, an observation that does raise the possibility that their presence represents a disease state. In another pyrotag study on honey bee gut communities in Arizona and Maryland, a few individuals and colonies showed higher incidence of sequences within 2 species of *Enterobacteriaceae* [14]. In comparison, the Mattila et al. [15] samples contain a greater variety of *Enterobacteriaceae* than the Arizona and Maryland samples, but a similar erratic distribution, which favors their proposal that these are pathogens.

Although a limited number of species or species groups make up the vast majority of the typical honey bee microbiota, each of these species is present as multiple strains, some of which may deserve species status. Some of this strain variation occurs within individual bees, and further variation can be found between colonies at different localities, based on longer reads of 16S rRNA [14]. Furthermore, strains within a species appear to undergo high rates of homologous recombination, at least for rRNA sequences in *Gilliamella apicola* and to a lesser extent in *Snodgrassella alvi* [14].

## Supporting Information

Table S1 **Pyrotag abundances for the 42 OTUs included in Mattila et al. (2012) dataset reanalysis and the Alpha-2 species group.**
(DOC)

Text S1 **Representative sequences for OTUs derived from community diversity reanalysis of Mattila et al. (2012) dataset (NCBI Sequence Read Archive #DRA000526).**
(DOC)

Text S2 **Summary of clustering and diversity analyses using alternative quality filter and clustering criteria.**
(PDF)
