# Supplementary material for: Independent Studies Using Deep Sequencing Resolve the Same Set of Core Bacterial Species Dominating Gut Communities of Honey Bees
Source: PLoS One. 2012 Jul 19;7(7):e41250. doi: 10.1371/journal.pone.0041250 (PMC3400611; doi:10.1371/journal.pone.0041250)
Supplement: Table S1 — Pyrotag abundances for the 42 OTUs included in Mattila et al. (2012) dataset reanalysis and the Alpha-2 species group. (DOC) [file pone.0041250.s001.doc]

**Supporting Information Table S1. Pyrotag abundances for 42 OTUs included in honeybee gut reanalysis and alpha-2 species group.**

| **OTU** | **PHYLOTYPE** | **M1** | **M2** | **M3** | **M4** | **M5** | **M6** | **M7** | **M8** | **M9** | **M10** | **M11** | **M12** | **S1** | **S2** | **S3** | **S4** | **S5** | **S6** | **S7** | **S8** | **S9** | **S10** |
| --- | --- | --- | --- | --- | --- | --- | --- | --- | --- | --- | --- | --- | --- | --- | --- | --- | --- | --- | --- | --- | --- | --- | --- |
| Cluster1 | gamma-1 | 1571 | 2049 | 662 | 1994 | 1972 | 2363 | 2548 | 3202 | 2268 | 1611 | 4682 | 1893 | 2405 | 1882 | 3011 | 2834 | 2765 | 1490 | 2134 | 1824 | 2192 | 2867 |
| Cluster30 | gamma-1 | 19 | 8 | 0 | 13 | 27 | 10 | 85 | 3 | 68 | 0 | 60 | 0 | 11 | 85 | 6 | 8 | 99 | 64 | 8 | 4 | 8 | 8 |
| Cluster140 | gamma-1 | 10 | 1 | 1 | 10 | 7 | 29 | 15 | 9 | 17 | 7 | 14 | 11 | 54 | 15 | 2 | 13 | 3 | 4 | 4 | 8 | 11 | 7 |
| Cluster227 | gamma-1 | 0 | 0 | 0 | 2 | 0 | 0 | 0 | 18 | 16 | 0 | 15 | 0 | 0 | 0 | 0 | 0 | 0 | 0 | 0 | 1 | 2 | 0 |
| Cluster1079 | gamma-1 | 4 | 6 | 0 | 5 | 0 | 0 | 1 | 3 | 3 | 0 | 9 | 7 | 10 | 2 | 9 | 6 | 1 | 0 | 2 | 6 | 9 | 7 |
| Cluster1406 | gamma-1 | 0 | 3 | 0 | 0 | 4 | 0 | 3 | 2 | 2 | 4 | 2 | 6 | 4 | 1 | 3 | 3 | 0 | 0 | 0 | 4 | 6 | 2 |
| Cluster1709 | gamma-1 | 2 | 0 | 0 | 2 | 0 | 0 | 2 | 1 | 3 | 0 | 2 | 1 | 3 | 2 | 3 | 2 | 0 | 0 | 0 | 1 | 2 | 2 |
| Cluster2475 | gamma-1 | 43 | 11 | 2 | 33 | 8 | 16 | 36 | 3 | 19 | 47 | 26 | 1 | 8 | 33 | 6 | 4 | 4 | 15 | 0 | 24 | 19 | 11 |
| Cluster173 | gamma-2 | 120 | 61 | 8 | 54 | 21 | 16 | 111 | 17 | 28 | 129 | 142 | 85 | 32 | 52 | 38 | 44 | 8 | 75 | 2 | 29 | 34 | 60 |
| Cluster16 | Bifidobacterium | 324 | 521 | 149 | 275 | 233 | 356 | 157 | 194 | 167 | 80 | 252 | 118 | 414 | 189 | 234 | 152 | 285 | 165 | 64 | 107 | 250 | 119 |
| Cluster46 | Bifidobacterium | 61 | 1 | 2 | 32 | 18 | 31 | 28 | 4 | 36 | 27 | 20 | 12 | 40 | 11 | 10 | 20 | 22 | 13 | 2 | 3 | 2 | 12 |
| Cluster2 | firm-5 | 170 | 344 | 44 | 471 | 316 | 248 | 722 | 395 | 431 | 320 | 1177 | 877 | 403 | 429 | 330 | 1341 | 220 | 474 | 454 | 428 | 341 | 492 |
| Cluster11 | firm-5 | 278 | 901 | 143 | 331 | 275 | 255 | 294 | 532 | 454 | 472 | 523 | 307 | 692 | 207 | 398 | 225 | 221 | 87 | 675 | 363 | 470 | 552 |
| Cluster33 | firm-5 | 241 | 250 | 37 | 209 | 182 | 106 | 121 | 104 | 175 | 138 | 337 | 191 | 419 | 106 | 199 | 39 | 10 | 214 | 285 | 84 | 162 | 290 |
| Cluster243 | firm-5 | 6 | 0 | 0 | 2 | 8 | 0 | 16 | 5 | 3 | 2 | 17 | 17 | 0 | 5 | 0 | 0 | 0 | 13 | 1 | 11 | 4 | 4 |
| Cluster5 | firm-4 | 898 | 570 | 151 | 419 | 524 | 366 | 549 | 487 | 321 | 334 | 1014 | 353 | 590 | 409 | 462 | 379 | 121 | 463 | 663 | 352 | 462 | 734 |
| Cluster26 | firm-4 | 27 | 45 | 0 | 37 | 33 | 44 | 46 | 64 | 32 | 54 | 92 | 125 | 43 | 47 | 31 | 7 | 10 | 23 | 66 | 34 | 87 | 79 |
| Cluster4 | beta | 148 | 221 | 29 | 191 | 269 | 77 | 369 | 182 | 366 | 778 | 792 | 831 | 382 | 281 | 260 | 387 | 49 | 152 | 920 | 123 | 498 | 183 |
| Cluster3852 | beta | 5 | 2 | 1 | 8 | 0 | 0 | 5 | 3 | 6 | 0 | 1 | 1 | 0 | 0 | 0 | 0 | 0 | 0 | 0 | 1 | 4 | 0 |
| Cluster208 | CFB-1 | 0 | 0 | 0 | 38 | 0 | 1 | 13 | 1 | 24 | 0 | 4 | 0 | 0 | 11 | 16 | 29 | 0 | 0 | 1 | 22 | 3 | 0 |
| Cluster20 | Enterobacteriaceae | 0 | 1 | 0 | 8 | 34 | 1 | 1 | 0 | 1 | 0 | 300 | 15 | 21 | 1 | 2 | 7 | 0 | 9 | 961 | 421 | 21 | 0 |
| Cluster746 | Enterobacteriaceae | 0 | 0 | 0 | 1 | 3 | 0 | 0 | 0 | 0 | 0 | 50 | 0 | 10 | 1 | 0 | 2 | 0 | 1 | 0 | 74 | 2 | 0 |
| Cluster1325 | Enterobacteriaceae | 0 | 0 | 0 | 3 | 7 | 1 | 0 | 0 | 5 | 0 | 1 | 0 | 0 | 0 | 1 | 0 | 0 | 0 | 0 | 1 | 0 | 1 |
| Cluster111 | Enterobacteriaceae | 0 | 96 | 0 | 8 | 56 | 4 | 0 | 0 | 2 | 0 | 20 | 0 | 1 | 3 | 21 | 1 | 2 | 0 | 3 | 68 | 8 | 2 |
| Cluster49 | Enterobacteriaceae | 0 | 0 | 0 | 168 | 170 | 1 | 1 | 5 | 0 | 0 | 3 | 0 | 11 | 11 | 4 | 38 | 0 | 0 | 0 | 5 | 11 | 7 |
| Cluster548 | Enterobacteriaceae | 0 | 2 | 0 | 6 | 4 | 0 | 4 | 0 | 1 | 0 | 5 | 3 | 1 | 5 | 1 | 1 | 0 | 0 | 1 | 13 | 2 | 1 |
| Cluster1671 | Enterobacteriaceae | 0 | 1 | 0 | 17 | 12 | 0 | 2 | 0 | 1 | 0 | 2 | 0 | 1 | 1 | 2 | 0 | 0 | 0 | 0 | 5 | 6 | 1 |
| Cluster3123 | Enterobacteriaceae | 1 | 0 | 0 | 2 | 0 | 0 | 4 | 0 | 2 | 0 | 2 | 1 | 0 | 0 | 0 | 0 | 0 | 0 | 0 | 2 | 2 | 0 |
| Cluster3680 | Enterobacteriaceae | 1 | 0 | 0 | 22 | 15 | 0 | 1 | 1 | 0 | 0 | 1 | 0 | 1 | 3 | 0 | 1 | 0 | 0 | 0 | 1 | 4 | 1 |
| Cluster7196 | Enterobacteriaceae | 0 | 0 | 0 | 5 | 2 | 0 | 0 | 0 | 0 | 0 | 0 | 0 | 1 | 1 | 0 | 0 | 0 | 0 | 0 | 2 | 0 | 0 |
| Cluster740 | Enterobacteriaceae | 0 | 0 | 0 | 3 | 4 | 0 | 0 | 0 | 0 | 0 | 0 | 0 | 1 | 0 | 0 | 0 | 0 | 0 | 0 | 0 | 0 | 0 |
| Cluster193 | Enterobacteriaceae | 1 | 0 | 0 | 3 | 4 | 19 | 19 | 46 | 1 | 0 | 97 | 0 | 0 | 4 | 0 | 0 | 0 | 1 | 0 | 1 | 19 | 0 |
| Cluster6040 | Enterobacteriaceae | 1 | 0 | 0 | 1 | 0 | 0 | 3 | 0 | 0 | 0 | 2 | 0 | 0 | 0 | 0 | 0 | 0 | 0 | 0 | 0 | 1 | 0 |
| Cluster119 | Melissococcus | 0 | 0 | 0 | 5 | 0 | 0 | 0 | 1 | 0 | 0 | 0 | 0 | 0 | 0 | 0 | 0 | 0 | 0 | 134 | 7 | 0 | 0 |
| Cluster273 | Enterobacteriaceae | 1 | 4 | 0 | 1 | 0 | 12 | 9 | 0 | 1 | 0 | 3 | 1 | 2 | 2 | 3 | 0 | 0 | 1 | 0 | 15 | 4 | 1 |
| Cluster1881 | Enterobacteriaceae | 0 | 0 | 0 | 0 | 0 | 0 | 1 | 0 | 1 | 0 | 4 | 0 | 0 | 2 | 0 | 0 | 0 | 0 | 0 | 9 | 0 | 0 |
| Cluster38 | Enterobacteriaceae | 17 | 139 | 1 | 243 | 388 | 27 | 54 | 9 | 45 | 1 | 67 | 0 | 31 | 228 | 49 | 58 | 2 | 1 | 48 | 577 | 244 | 20 |
| Cluster195 | Enterobacteriaceae | 0 | 0 | 0 | 0 | 0 | 0 | 0 | 0 | 0 | 0 | 157 | 0 | 0 | 0 | 0 | 0 | 0 | 0 | 0 | 0 | 2 | 0 |
| Cluster6128 | Enterobacteriaceae | 0 | 0 | 0 | 2 | 2 | 0 | 0 | 0 | 0 | 0 | 0 | 0 | 2 | 0 | 1 | 0 | 0 | 0 | 1 | 1 | 0 | 0 |
| Cluster544 | Lactobacillus_kunkeei | 1 | 2 | 0 | 0 | 1 | 1 | 0 | 0 | 1 | 8 | 1 | 0 | 2 | 1 | 2 | 0 | 0 | 0 | 1 | 1 | 1 | 0 |
| Cluster156 | Uncultured bacterium | 0 | 0 | 0 | 0 | 2 | 0 | 0 | 0 | 0 | 1 | 36 | 0 | 3 | 21 | 0 | 18 | 8 | 0 | 0 | 3 | 1 | 6 |
| Cluster4293 | Uncultured bacterium | 0 | 0 | 0 | 0 | 2 | 0 | 0 | 0 | 0 | 0 | 2 | 0 | 0 | 1 | 0 | 0 | 0 | 0 | 0 | 3 | 1 | 0 |
|  | **TOTAL READS** | **3950** | **5239** | **1230** | **4624** | **4603** | **3984** | **5220** | **5291** | **4500** | **4013** | **9934** | **4856** | **5598** | **4052** | **5104** | **5619** | **3830** | **3265** | **6430** | **4638** | **4895** | **5469** |
|  |  |  |  |  |  |  |  |  |  |  |  |  |  |  |  |  |  |  |  |  |  |  |  |
| **OTU** | **PHYLOTYPE** | **M1** | **M2** | **M3** | **M4** | **M5** | **M6** | **M7** | **M8** | **M9** | **M10** | **M11** | **M12** | **S1** | **S2** | **S3** | **S4** | **S5** | **S6** | **S7** | **S8** | **S9** | **S10** |
| Cluster1-alpha | alpha-2 | 54 | 58 | 1 | 43 | 25 | 34 | 16 | 124 | 110 | 107 | 69 | 23 | 90 | 60 | 54 | 102 | 59 | 84 | 19 | 19 | 97 | 21 |
