# Supplementary material for: Independent Studies Using Deep Sequencing Resolve the Same Set of Core Bacterial Species Dominating Gut Communities of Honey Bees
Source: PLoS One. 2012 Jul 19;7(7):e41250. doi: 10.1371/journal.pone.0041250 (PMC3400611; doi:10.1371/journal.pone.0041250)
Supplement: Text S1 — Representative sequences for OTUs derived from community diversity reanalysis of Mattila et al. (2012) dataset (NCBI Sequence Read Archive #DRA000526). (DOC) [file pone.0041250.s002.doc]

**Supporting Information Text 1. Representative sequences for OTUs derived from community diversity re-analysis of singly/multiply-mated honeybee gut pyrosequencing dataset (NCBI Sequence Read Archive #DRA000526).**

Sequencing adapters and barcodes have been trimmed from all sequences.

>Cluster1 [Species group=Gilliamella] [Pyrotag=GPEDTIY04JG3TQ]

AGAGTTTGATCCTGGCTCAGATTGAACGCTGGCGGCAGGCTTAACACATGCAAGTCGAACGGTAACATGAGTGCTTGCACTTGATGACGAGTGGCGGACGGGTGAGTAAAGTATGGGGATCTGCCGAATGGAGGGGGACAACAGTTGGAAACGACTGCTAATACCGCATAAAGTTGAGAGACCAAAGCATGGGACTTACGGGCCATGCGCCATTCGATGAACCCATATGGGATTAGCTAGTTGGTAGGGTAATGGCTTACCAAGGCGACGATCTCTAGCTGGTCTGAGAGGATGACCAGCCACACTGGAACTGAGACACGGTCCAGACTCCTACGGGAGGCAGC

>Cluster2 [Species group=Firm5] [Pyrotag=GPEDTIY04I420C]

AGAGTTTGATCATGGCTCAGGACGAACGCTGGCGGCGTGCCTAATACATGCAAGTCGAGCGAGCAAAGTTAAAGGAATACTTCGGTAGGAATTTAATAGCGCGAGCGGCGGATGGGTGAGTAACACGTGGGCAACCTGCCCTTTAGCTTGGGATACCACTTGGAAACAGGTGCTAATACCAAATAAGAAGTAAGAGCGCATGCTCAAGCTATGAAAGGCGGCTTTCGAGCTGTCACTAAAGGATGGGCCCGCGGTGCATTAGCTAGTTGGTAAGGTAACGGCTTACCAAGGCAATGATGCATAGCCGAGTTGAGAGACTGATCGGCCACATTGGGACTGAGACACGGCCAAACCTCCTACGGGAGGCTGC

>Cluster4 [Species group=Beta] [Pyrotag=GPEDTIY04H7XCI]

AGAGTTTGATCATGGCTCAGATTGAACGCTGGCGGCATGCTTTACACATGCAAGTCGAACGGCAGCACGGAGAGCTTGCTCTCTGGTGGCGAGTGGCGAACGGGTGAGTAATGCATCGGAACGTACCGAGTAATGGGGGATAACTGTCCGAAAGGATGGCTAATACCGCATACGCCCTGAGGGGGAAAGCGGGGGATCGAAAGACCTCGCGTTATTTGAGCGGCCGATGTTGGATTAGCTAGTTGGTGGGGTAAAGGCCTACCAAGGCGACGATCCATAGCGGGTCTGAGAGGATGATCCGCCACATTGGGACTGAGACACGGCCCAAACTCCTACGGGAGGCAGC

>Cluster5 [Species group=Firm4] [Pyrotag=GPEDTIY04I9NZ1]

AGAGTTTGATCATGGCTCAGGACGAACGCTGGCGGCGTGCCTAATACATGCAAGTCGAGCGCGGGAAGTCAGGGAAGCCTTCGGGTGGAACTGGTGGAACGAGCGGCGGATGGGTGAGTAACACGTAGGTAACCTGCCCTAAAGCGGGGGATACCATCTGGAAACAGGTGCTAATACCGCATAACCCCAGCAGTCACATGAGTGCTGGTTGAAAGACGGCTTCGGCTGTCACTTTAGGATGGACCTGCGGCGTATTAGCTAGTTGGTGGAGTAACGGTTCACCAAGGCGATGATACGTAGCCGACCTGAGAGGGTAATCGGCCACATTGGGACTGAGACACGGCCCAAACTCCTACGGGAGGCTGC

>Cluster11 [Species group=Firm5] [Pyrotag=GPEDTIY04INVU8]

AGAGTTTGATCCTGGCTCAGGACGAACGCTGGCGGCGTGCCTAATACATGCAAGTCGAGCGAGCAATTTTGACGGAATACTTCGGTAGGAAGTCAGAAGCGCGAGCGGCGGATGGGTGAGTAACACGTGGGCAACCTGCCCTTTAGCTTGGGATACCACTTGGAAACAGGTGCTAATACCAAATAAGCAGCAAGAGCGCATGCTCAAGCTGAGAAAGGCGGCTTTCGAGCTGTCACTAAAGGATGGGCCCGCGGTGCATTAGCTAGTTGGTAAGGTAACGGCCTACCAAGGCGATGATGCATAGCCGAGTTGAGAGACTGACCGGCCACATTGGGACTGAGACACGGCCCAAACTCCTACGGGAGGCAGC

>Cluster16 [Species group=Bifido] [Pyrotag=GPEDTIY04IR3F9]

AGAGTTTGATCCTGGCTCAGGATGAACGCTGGCGGCGTGCTTAACACATGCAAGTCGAACGGGATCCGGGCAGCTTGCTGCCTGGTGAGAGTGGCGAACGGGTGAGTAATGCGTGACCAACCTGCCCCATGCTTCGGAATAGCTCCTGGAAACGGGTGGTAATGCCGGATGCTCCGCACTGTCGCATGATGGTGTGGGAAAGGGTTTACCGGCATGGGATGGGGTCGCGTCCTATCAGCTTGTTGGCGGGGTGATGGCCTGCCAAGGCTTCGACGGGTAGCCGGCCTGAGAGGGCGACCGGCCACATTGGGACTGAGATACGGCCCAGACTCCTACGGGAGGCAGC

>Cluster20 [Species group=Enterobacteriaceae] [Pyrotag=GPEDTIY04IAV8B]

AGAGTTTGATCATGGCTCAGATTGAACGCTGGCGGCAGGCCTAACACATGCAAGTCGAGCGGCAGCGGAAAGAAGCTTGCTTCTTTGCCGGCGAGCGGCGGACGGGTGAGTAATGTCTGGGGATCTGCCCGATGGAGGGGGATAACCACTGGAAACGGTGGCTAATACCGCATAACGTCGCAAGACCAAAGTGGGGGACCTTCGGGCCTCACGCCATAGGATGAACCCAGATGGGATTAGCTAGTAGGTGGGGTAAAGGCTCACCTAGGCGACGATCTCTAGCTGGTCTGAGAGGATGACCAGCCACACTGGAACTGAGACACGGTCCAGACTCCTACGGGAGGCAGC

>Cluster26 [Species group=Firm4] [Pyrotag=GPEDTIY04JEHLF]

AGAGTTTGATCATGGCTCAGGACGAACGCTGGCGGCGTGCCTAATACATGCAAGTCGAGCGAGCGAGATGAACGGAATACTTCGGTAGGAAGTTCAGATTGCGAGCGGCGGATGGGTGAGTAACACGTAGGCAATCTACCCTTAAGCGGGGGATAACACCTGGAAACAGGTGCTAATACCGCATAAAACTAAGGACGCCTGTTCTAAAGTTAAAAGACGGTGAGAGCTGTCACTTAAGGATGAGCCTGCGGCGTATTAGCTAGTTGGCAAGGTAAGAGCTTACCAAGGCGATGATACGTAGCCGACCTGAGAGGGTAATCGGCCACATTGGGACTGAGACACGGCCCAAACTCCTACGGGAGCTGC

>Cluster30 [Species group=Gilliamella] [Pyrotag=GPEDTIY04I63K7]

AGAGTTTGATCATGGCTCAGATTGAACGCTGGCGGCAGGCTTAACACATGCAAGTCGAACGGTAACATGAGTGCTTGCACTTGATGACGAGTGGCGGACGGGTGAGTAAGGTATGGGGATCTGCCGAATGGAGAGGGACAACAGTTGGAAACGACTGCTAATACCTCATAAAGTTGAGAGACCAAAGCGTGGGACCTTCGGGCCACGTGCCATTTGATGAACCCATATGGGATTAGCTAGTTGGTGGGGTAATAGCTCACCAAGGCGACGATCTCTAGCTGGTCTGAGAGGATGACCAGCCACACTGGAACTGAGACACGGTCCAGACTCCTACGGGAGGCTGC

>Cluster33 [Species group=Firm5] [Pyrotag=GPEDTIY04ISJ49]

AGAGTTTGATCATGGCTCAGGACGAACGCTGGCGGCGTGCCTAATACATGCAAGTCGAGCGAGCAATCTTAACGGAATACTTCGGTAGGAAGTCAAGAGCGCGAGCGGCGGATGGGTGAGTAACACGTGGGCAACCTGCCCTATAGCTTGGGATACCACTTGGAAACAGGTGCTAATACCAAATAAGCAGCACGAGCGCATGCTCAAGCTGTAAAAGGCGGCTTTAAGCTGTCACTAAAGGATGGGCCCGCGGTGCATTAGCTAGTTGGCAAGGTAACGGCCTACCAAGGCGATGATGCATAGCCGAGTTGAGAGACTGAACGGCCACATTGGGACTGAGACACGGCCCAAACTCCTACGGGAGGCTGC

>Cluster38 [Species group=Enterobacteriaceae] [Pyrotag=GPEDTIY04JLQN4]

AGAGTTTGATCCTGGCTCAGATTGAACGCTGGCGGCAGGCCTAACACATGCAAGTCGAGCGGTAGCACAGAGAGCTTGCTCTCGGGTGACGAGCGGCGGACGGGTGAGTAATGTCTGGGAAACTGCCTGATGGAGGGGGATAACTACTGGAAACGGTAGCTAATACCGCATAACGTCGCAAGACCAAAGTGGGGGACCTTCGGGCCTCATGCCATCAGATGTGCCCAGATGGGATTAGCTAGTAGGTGGGGTAACGGCTCACCTAGGCGACGATCCCTAGCTGGTCTGAGAGGATGACCAGCCACACTGGAACTGAGACACGGTCCAGACTCCTACGGGAGGCAGC

**Supporting Information Text 1. Representative sequences for OTUs derived from community diversity re-analysis of singly/multiply-mated honeybee gut pyrosequencing dataset (NCBI Sequence Read Archive #DRA000526).**

>Cluster46 [Species group=Bifido] [Pyrotag=GPEDTIY04H5O4Q]

AGAGTTTGATCATGGCTCAGGATGAACGCTGGCGGCGTGCTTAACACATGCAAGTCGAACGGGATCCAGGCAGCTTGCTGTCTGGTGAGAGTGGCGAACGGGTGAGTAATGCGTGACCAACCTGCCCCATACTCCGGAATAGCTCCTGGAAACGGGTGGTAATGCCGGGTGTTCCGCATCATCGCATGATGGTGTGGGAAAGGGTTTACCGGTATGGGATGGGGTCGCGTCCTATCAGCTTGTTGGTGGGGTGATGGCCTACCAAGGCTTCGACGGGTAGCCGGCCTGAGAGGGCGACCGGCCACATTGGGACTGAGATACGGCCCAGACTCCTACGGGAGGCTGC

>Cluster49 [Species group=Enterobacteriaceae] [Pyrotag=GPEDTIY04H7G6B]

GAGTTTGATCATGGCTCAGATTGAACGCTGGCGGCAGGCCTAACACATGCAAGTCGAGCGGCAGCGGAAAGTAGCTTGCTACTTTGCCGGCGAGCGGCGGACGGGTGAGTAATGTCTGGGAAACTGCCTGATGGAGGGGGATAACTACTGGAAACGGTAGCTAATACCGCATAACGTCGCAAGACCAAAGAGGGGGACCTTCGGGCCTCTTGCCATCAGATGTGCCCAGATGGGATTAGCTAGTAGGTGGGGTAACGGCTCACCTAGGCGACGATCCCTAGCTGGTCTGAGAGGATGACCAGCCACACTGGAACTGAGACACGGTCCAGACTCCTACGGGAGGCAGC

>Cluster111 [Species group=Enterobacteriaceae] [Pyrotag=GPEDTIY04IGZOK]

AGAGTTTGATCCTGGCTCAGATTGAACGCTGGCGGCAGGCCTAACACATGCAAGTCGAACGGTAGCACAGAGGAGCTTGCTCCTCGGGTGACGAGTGGCGGACGGGTGAGTAATGTCTGGGAAACTGCCCGATGGAGGGGGATAACTACTGGAAACGGTAGCTAATACCGCATAATGTCGCAAGACCAAAGAGGGGGACCTTCGGGCCTCTTGCCATCGGATGTGCCCAGATGGGATTAGCTAGTAGGTGGGGTAATGGCTCACCTAGGCGACGATCCCTAGCTGGTCTGAGAGGATGACCAGCCACACTGGAACTGAGACACGGTCCAGACTCCTACGGGAGGCAGC

>Cluster119 [Species group=Melissococcus] [Pyrotag=GPEDTIY04I4Y40]

AGAGTTTGATCATGGCTCAGGACGAACGCTGGCGGCGTGCCTAATACATGCAAGTCGAACGCTTCTTCTGATTAAGCTTGCTTAAGAGGAAAGAAGAGTGGCGGACGGGTGAGTAACACGTGGGCAACCTGCCCATCAGAAGGGGATAACATTTGGAAACAGGTGCTAATACCGTATAAGACTTTTTTTCGCATGAAGAGGAGTTAAAAGGCGCTTTCGGGTGTCACTGATGGATGGGCCCGCGGTGCATTAGCTAGTTGGTGGGGTAAAGGCTCACCAAGGCAACGATGCATAGCCGACCTGAGAGGGTGATCGGCCACACTGGGACTGAGACACGGCCCAGACTCCTACGGGAGGCTGC

>Cluster140 [Species group=Gilliamella] [Pyrotag=GPEDTIY04IBVBA]

AGAGTTTGATCCTGGCTCAGATTGAACGCTGGCGGCAGGCTTAACACATGCAAGTCGAACGGTAACATGAGTGCTTGCACTTGATGACGAGTGGCGGACGGGTGAGTAAAGTATGGGGATCTGCCGAATGGAAGGGGACAACAGTTGGAAACGACTGCTAATACCGTATAATGTCGCGAGACCAAAGCATGGGACTTTCGGGCCATGCGCCATTTGATGAACCCATATGGGATTAGCTAGTAGGTGGGGTAAAGGCTCACCTAGGCGACGATCTCTAGCTGGTCTGAGAGGATGGCCAGCCACACTGGAACTGAGACACGGTCCAGACTCCTACGGGAGGCAGC

>Cluster156 [Species group=Enterobacteriaceae] [Pyrotag=GPEDTIY04I8HJD]

AGAGTTTGATCATGGCTCAGATTGAACGCTGGCGGCAGGCCTAACACATGCAAGTCGAGCGGTAGCACAAGAGAGCTTGCTCTCTGGGTGACGAGCGGCGGACGGGTGAGTAATGTCTGGGAAACTGCCTGATGGAGGGGGATAACTACTGGAAACGGTAGCTAATACCGCATGACGTCTTCGGACCAAAGTGGGGGACCTTCGGGCCTCACGCCATCAGATGTGCCCAGATGGGATTAGCTAGTAGGTGGGGTAATGGCTCACCTAGGCGACGATCTCTAGCTGGTCTGAGAGGATGACCAGCCACACTGGAACTGAGACACGGTCCAGACTCCTACGGGAGGCTGC

>Cluster173 [Species group=Gamma2] [Pyrotag=GPEDTIY04I94MA]

AGAGTTTGATCATGGCTCAGATTGAACGCTGGCGGCAGGCTTAACACATGCAAGTCGAACGGTAACATGAGTGCTTGCACTTGATGACGAGTGGCGGACGGGTGAGTAATGTATGGGGATCTGCCGAATGGATTGGGACAACAGTTGGAAACGACTGCTAATACCGAATAATGTTGCGAGACCAAAGGGTGCTTACGGGCACTTGCCATTTGATGAACCCATATGAGATTAGCTAGTTGGTGGGGTAAAGGCTCACCAAGGCGACGATCTCTAGCTGGTCTGAGAGGATGACCAGCCACACTGGAACTGAGACACGGTCCAGACTCCTACGGGAGGCTAGC

>Cluster193 [Species group=Enterobacteriaceae] [Pyrotag=GPEDTIY04I2FNU]

AGAGTTTGATCATGGCTCAGATTGAACGCTGGCGGCAGGCCTAACACATGCAAGTCGAACGGTAACAGAAAGAAGCTTGCTTCTTTGCTGACGAGTGGCGGACGGGTGAGTAATGTCTGGGAAACTGCCCGATGGAGGGGGATAACTACTGGAAACGGTAGCTAATACCGCATAACGTCGCAAGACCAAAGAGGGGGACCTTCGGGCCTCTTGCCATCGGATGTGCCCAGATGGGATTAGCTAGTAGGCGGGGTAACGGCCCACCTAGGCGACGATCTCTAGCTGGTCTGAGAGGATGACCAGCCACACTGGAACTGAGACACGGTCCAGACTCCTACGGGAGGCTGC

>Cluster195 [Species group=Enterobacteriaceae] [Pyrotag=GPEDTIY04IKTMX]

AGAGTTTGATCCTGGCTCAGATTGAACGCTGGCGGCAGGCCTAACACATGCAAGTCGAGCGGCAGCGGGAGGAAGCTTGCTTCCTCGCCGGCGAGCGGCGGACGGGTGAGTAATGTCTGGGAAACTGCCCGATGGAGGGGGATAACTACTGGAAACGGTAGCTAATACCGCATAACGTCGCAAGACCAAAGTGGGGGACCTTAGGGCCTCACACCATCGGATGTGCCCAGATGGGATTAGCTAGTAGGTGGGGTAATGGCTCACCTAGGCGACGATCCCTAGCTGGTCTGAGAGGATGACCAGCCACACTGGAACTGAGACACGGTCCAGACTCCTACGGGAGGCAGC

>Cluster208 [Species group=CFB-1] [Pyrotag=GPEDTIY04JDB8F]

AGAGTTTGATCCTGGCTCAGGATGAACGCTAGCGGGAGGCCTAACACATGCAAGCCGAGGGGTATTTTGGAGCTTGCTTCAAGAGAGACCGGCGTACGGGTGCGTAACGCGTATGCAACTTACCTGCGTCACTGGGATAGCCCGGGGAAACTCGGATTAATACCGGATGAAATACGAGGCGGCATTGCTTTGTATTAAAAGATTTATCGGAAGCAGATAGGCATGCGTAGGATTAGTTAGTTGGTAAGGTAACGGCTTACCAAGACGATGATCCTTAGGGGGCCTGAGAGGGTGAACCCCCACACTGGAACTGAGACACGGACCAGACTCCTACGGGAGGCAGC

>Cluster227 [Species group=Gilliamella] [Pyrotag=GPEDTIY04I1TSC]

GAGAGTTTGATCCTGGCTCAGATTGAACGCTGGCGGCAGGCTTAACACATGCAAGTCGAACGGTAACATGAGTGCTTGCACTTGATGACGAGTGGCGGACGGGTGAGTAATGTATGGGGATCTGCCAGATGGAGGGGGACAACAGTTGGAAACGACTGCTAATACCGCATAAAGTTGAGAGACCAAAGTACGGTTCCGCGAGGACGTGCGCCATTTGATGAACCCATATGGGATTAGCTAGTTGGTGGGGTAAAGGCTTACCAAGGCGACGATCTCTAGCTGGTCTGAGAGGATGACCAGCCACACTGGAACTGAGACACGGTCCAGACTCCTACGGGAGGCAGC

>Cluster243 [Species group=Firm5] [Pyrotag=GPEDTIY04IVIUZ]

AGAGTTTGATCATGGCTCAGGACGAACGCTGGCGGCGTGCCTAATACATGCAAGTCGAGCGAGGGAAGGACATGAATTTTTCGGAAGGATTGTTTAGACCGAGCGGCGGATGGGTGAGTAACACGTAGGGAACCTGCCAAACAGACGGGGATACCACTTGGAAACAAGTGCTAATACCGGATAGAGCACTTTATCGCATGATAGAGTGAGGAAAGGGCGGCGAAAGCTGTCGCTGATTGATGGACCTGCGGCGTATTAGCTAGTTGGGGAGGTAAAGGCTCACCAAGGCGATGATACGTAGCCGACCTGAGAGGGTAAACGGCCACATTGGGACTGAGACACGGCCCAAACTCCTACGGGAGCNGC

**Supporting Information Text 1. Representative sequences for OTUs derived from community diversity re-analysis of singly/multiply-mated honeybee gut pyrosequencing dataset (NCBI Sequence Read Archive #DRA000526).**

>Cluster273 [Species group=Enterobacteriaceae] [Pyrotag=GPEDTIY04JUOOR]

AGAAGTTTGATCATGGCTCAGATTGAACGCTGGCGGCAGGCCTAACACATGCAAGTCGGACGGTAGCACAGAGAGCTTGCTCTTGGGTGACGAGTGGCGGACGGGTGAGTAATGTCTGGGGATCTGCCCGATAGAGGGGGATAACCACTGGAAACGGTGGCTAATACCGCATAACGTCGCAAGACCAAAGAGGGGGACCTTCGGGCCTCTCACTATCGGATGAACCCAGATGGGATTAGCTAGTAGGCGGGGTAATGGCCCACCTAGGCGACGATCCCTAGCTGGTCTGAGAGGATGACCAGCCACACTGGAACTGAGACACGGTCCAGACTCCTACGGGAGGCTGC

>Cluster544 [Species group=Lactobacillus kunkeei] [Pyrotag=GPEDTIY04H1F18]

AGAGTTTGATCATGGCTCAGGACGAACGCTGGCGGCGTGCCTAATACATGCAAGTCGAACGAGCTCTCCCAAATTGATTTTATGCTTGCATAAATGATTTTTGGATTCGGAGCGAGTGGCGAACTGGTGAGTAACACGTGGGTAACCTGCCCCGAAGCGGGGGATAACATTTGGAAACAAGTGCTAATACCGCATAATTAGTTGGAACCGCATGGTTCCAACTTGAAAGATGGCTCTGCTATCACTTTGGGATGGACCCGCGCCGTATTAGTTAGTTGGTGAGATAAAAGCCCACCAAGACGATGATACGTAGCCGACCTGAGAGGGTAATCGGCCACATTGGGACTGAGACACGGCCCAGACTCCTACGGGAGGCAGC

>Cluster548 [Species group=Enterobacteriaceae] [Pyrotag=GPEDTIY04IO8RZ]

AGAGTTTGATCATGGCTCAGATTGAACGCTGGCGGCAGGCTTAACACATGCAAGTCGAACGGTAACATGAGTGCTTGCACTTGATGACGAGTGGCGGACGGGTGAGTAATGTCTGGGAAACTGCCTGATGGAGGGGGATAACTACTGGAAACGGTAGCTAATACCGCATAACGTCGCAAGACCAAAGAGGGGGACCTTCGGGCCTCTTGCCATCAGATGTGCCCAGATGGGATTAGCTAGTAGGTGGGGTAACGGCTCACCTAGGCGACGATCCCTAGCTGGTCTGAGAGGATGACCAGCCACACTGGAACTGAGACACGGTCCAGACTCCTACGGGAGGCAGC

>Cluster740 [Species group=Enterobacteriaceae] [Pyrotag=GPEDTIY04JDHQT]

AGAGTTTGATCATGGCTCAGATTGAACGCTGGCGGCAGGCCTAACACATGCAAGTCGAACGGCAGCACAGAAGAGCTTGCTCTTTGGGTGGCGAGTGGCGGACGGGTGAGTAATGTCTGGGAAACTGCCCGATGGAGGGGGATAACTACTGGAAACGGTAGCTAATACCGCATAACGTCGCGAGACCAAAGTGGGGGACCTTCGGGCCTCACACCATCGGATGTGCCCAGATGGGATTAGCTAGTAGGTGGGGTAATGGCTCACCTAGGCGACGATCCCTAGCTGGTCTGAGAGGATGACCAGCCACACTGGAACTGAGACACGGTCCAGACTCCTACGGGAGCAGC

>Cluster746 [Species group=Enterobacteriaceae] [Pyrotag=GPEDTIY04JZNTW]

AGAGTTTGATCCTGGCTCAGATTGAACGCTGGCGGCAGGCCTAACACATGCAAGTCGAGCGGCAGCGGAAGGAAGCTTGCTTCCTTGCCGGCGAGCGGCGGACGGGTGAGTAATGTCTGGGGATCTGCCCGATGGAGGGGGATAACTACTGGAAACGGTAGCTAATACCGCATAACGTCGCAAGACCAAAGTGGGGGACCTTCGGGCCTCACACCATCGGATGAACCCAGATGGGATTAGCTAGTTGGTAGGGTAATGGCTTACCAAGGCGACGATCTCTAGCTGGTCTGAGAGGATGACCAGCCACACTGGAACTGAGACACGGTCCAGACTCCTACGGGAGGCTGC

>Cluster1079 [Species group=Gilliamella] [Pyrotag=GPEDTIY04I9RT7]

AGAGTTTGATCCTGGCTCAGGACGAACGCTGGCGGCGTGCCTAATACATGCAAGTCGAGCGCGGGAAGTCAGGGAAGCCTTCGGGTGGAACTGGTGGAACGAGCGGCGGATGGGTGAGTAACACGTAGGTAACCTGCCCTAAAGCGGGGGATACCATCTGGAAACAGGTGCTAATACCGCATAAAGTTGAGAGACCAAAGCATGGGACTTACGGGCCATGCGCCATTCGATGAACCCATATGGGATTAGCTAGTTGGTAGGGTAATGGCTTACCAAGGCGACGATCTCTAGCTGGTCTGAGAGGATGACCAGCCACACTGGAACTGAGACACGGTCCAGACTCCTACGGGAGCTGC

>Cluster1325 [Species group=Enterobacteriaceae] [Pyrotag=GPEDTIY04JHMPB]

AGAGTTTGATCCTGGCTCAGATTGAACGCTGGCGGCAGGCCTAACACATGCAAGTCGGGCGGTAACACAGGGAGCTTGCTCCTGGGTGACGAGCGGCGGACGGGTGAGTAATGTCTGGGAAACTGCCCGATGGAGGGGGATAACTACTGGAAACGGTAGCTAATACCGCATAACGTCTTCGGACCAAAGAGGGGGACCTTCGGGCCTCTTGCCATCGGATGTGCCCAGATGGGATTAGCTAGTAGGTGGGGTAATGGCTCACCTAGGCGACGATCCCTAGCTGGTCTGAGAGGATGACCAGCCACACTGGAACTGAGACACGGTCCAGACTCCTACGGGAGGCAGC

>Cluster1406 [Species group=Gilliamella] [Pyrotag=GPEDTIY04JS6E6]

AGAGTTTGATCATGGCTCAGATTGAACGCTGGCGGCAGGCTTAACACATGCAAGTCGAACGGTAACATGAGTGCTTGCACTTGATGACGAGTGGCGGACGGGTGAGTAAAGTATGGGGATCTGCCGAATGGAGGGGGACAACAGTTGGAAACGACTGCTAATACCGCATAAAGTTGAGAGACCAAAGCATGGGACTTACGGGCCATGCGCCATTCGATGAACCCATATGGGATTAGCTAGTTGGTGGGGTAAATGCCTACCAAGGCGACGATCCATAGCGGGTCTGAGAGGATGATCCGCCACATTGGGACTGAGACACGGCCCAAACTCCTACGGGAGGCTGC

>Cluster1671 [Species group=Enterobacteriaceae] [Pyrotag=GPEDTIY04IVFW6]

AGAGTTTGATCATGGCTCAGATTGAACGCTGGCGGCAGGCCTAACACATGCAAGTCGAGCGGTAACACAGGGAGCTTGCTCCTGGGTGACGAGCGGCGGACGGGTGAGTAATGTCTGGGAAACTGCCTGATGGAGGGGGATAACTACTGGAAACGGTAGCTAATACCGCATAACGTCGCAAGACCAAAGAGGGGGACCTTCGGGCCTCTTGCCATCAGATGTGCCCAGATGGGATTAGCTAGTTGGTAGGGTAAAGGCTTACCAAGGCGACGATCTCTAGCTGGTCTGAGAGGATGACCAGCCACACTGGAACTGAGACACGGTCCAGACTCCTACGGGAGGCTGC

>Cluster1709 [Species group=Gilliamella] [Pyrotag=GPEDTIY04JGY6B]

AGAGTTTGATCATGGCTCAGATTGAACGCTGGCGGCATGCTTTACACATGCAAGTCGAACGGCAGCACGGAGAGCTTGCTCTCTGGTGGCGAGTGGCGAACGGGTGAGTAATGCATCGGAACGTACCGAGTAATGGGGGATAACTGTCCGAAAGGATGGCTAATACCGCATAACGTTGAGAGACCAAAGCATGGGACCTTCGGGCCATGCGCCATTTGATGAACCCATATGGGATTAGCTAGTTGGTAGGGTAAAGGCTTACCAAGGCGACGATCTCTAGCTGGTCTGAGAGGATGACCAGCCACACTGGAACTGAGACACGGTCCAGACTCCTACGGGAGGCTGC

>Cluster1881 [Species group=Enterobacteriaceae] [Pyrotag=GPEDTIY04IZPPV]

AGAGTTTGATCCTGGCTCAGATTGAACGCTGGCGGCAGGCCTAACACATGCAAGTCGGACGGTAGCACAGAGGAGCTTGCTCCTCGGGTGACGAGTGGCGGACGGGTGAGTAATGTCTGGGGATCTGCCCGATGGAGGGGGATAACTACTGGAAACGGTAGCTAATACCGCATAACGTCGCAAGACCAAAGTGGGGGACCTTCGGGCCTCACACCATCGGATGAACCCAGATGGGATTAGCTAGTAGGCGGGGTAAAGGCCCACCTAGGCGACGATCTCTAGCTGGTCTGAGAGGATGACCAGCCACACTGGAACTGAGACACGGTCCAGACTCCTACGGGAGGCAGC

**Supporting Information Text 1. Representative sequences for OTUs derived from community diversity re-analysis of singly/multiply-mated honeybee gut pyrosequencing dataset (NCBI Sequence Read Archive #DRA000526).**

>Cluster2475 [Species group=Gilliamella] [Pyrotag=GPEDTIY04JUZTN]

AGAGTTTGATCCTGGCTCAGGACGAACGCTGGCGGCGTGCCTAATACATGCAAGTCGAGCGCGGGAAGTCAGGGAAGCCTTCGGGTGGAACTGGTGGAACGAGCGGCGGACGGGTGAGTAAGGTATGGGGATCTGCCGAATGGAGGGGGACAACAGTTGGAAACGACTGCTAATACCGCATAATGTTGAGAAACCAAAGCATGGGACCTTCGGGCCATGCGCCATTTGATGAACCCATATGGGATTAGCTAGTTGGTAGGGTAAAGGCTTACCAAGGCGACGATCTCTAGCTGGTCTGAGAGGATGACCAGCCACACTGGAACTGAGACACGGTCCAGACTCCTACGGGAGGCTGC

>Cluster3123 [Species group=Enterobacteriaceae] [Pyrotag=GPEDTIY04JSLJO]

AGAGTTTGATCCTGGCTCAGATTGAACGCTGGCGGCAGGCCTAACACATGCAAGTCGAACGGTAACAGGAAGCAGCTTGCTGCTTCGCTGACGAGTGGCGGACGGGTGAGTAATGTCTGGGAAACTGCCTGATGGAGGGGGATAACTACTGGAAACGGTAGCTAATACCGCATAATGTCGCAAGACCAAAGAGGGGGACCTTCGGGCCTCTTGCCATCAGATGTGCCCAGATGGGATTAGCTAGTAGGTGGGGTAACGGCGTCACCTAGGCGACGATCCCTAGCTGGTCTGAGAGGATGACCAGCCACACTGGAACTGAGACACGGTCCAGACTCCTACGGAGGCTGC

>Cluster3680 [Species group=Enterobacteriaceae] [Pyrotag=GPEDTIY04IN9AV]

AGAGTTTGATCATGGCTCAGATTGAACGCTGGCGGCAGGCCTAACACATGCAAGTCGAGNGNCANAGGAGCTTGCTCCTGGGTGACGAGNGGCGGACGGGTGAGTAATGTCTGGGAAACTGCCTGATGGAGGGGGATAACTACTGGAAACGGTAGCTAATACCGCATAACGTCGCAAGACCAAAGAGGGGGACCTTCGGGCCTCTTGCCATCAGATGTGCCCAGATGGGATTAGCTAGTAGGTGGGGTAACGGCTCACCTAGGCGACGATCCCTAGCTGGTCTGAGAGGATGACCAGCCACACTGGAACTGAGACACGGTCCAGACTCCTACGGGAGGCAGC

>Cluster3852 [Species group=Beta] [Pyrotag=GPEDTIY04JA3IH]

AGAGTTTGATCCTGGCTCAGATTGAACGCTGGCGGCAGGCCTAACACATGCAAGTCGAACGGTAGCACAGAGAGCTTGCTCTCTGGTGGCGAGTGGCGAACGGGTGAGTAATGCATCGGAACGTACCGAGTAATGGGGGATAACTGTCCGAAAGGATGGCTAATACCGCATACGCCCTGAGGGGGAAAGCGGGGGATCTTAGTGACCTCGCGTTATTTGAGCGGCCGATGTTGGATTAGCTGGTTGGTGGGGTAAAGGCCTACCAAGGCGACGATCCATAGCGGGTCTGAGAGGATGATCCGCCACATTGGGACTGAGACACGGCCCAAACTCCTACGGGAGGCTGC

>Cluster4293 [Species group=Enterobacteriaceae] [Pyrotag=GPEDTIY04IDIFD]

AGAGTTTGATCATGGCTCAGATTGAACGCTGGCGGCAGGCCTAACACATGCAAGTCGAGCGGTAGCACAGAGAGCTTGCTCTCGGGTGACGAGCGGCGGACGGGTGAGTAATGTCTGGGAAACTGCCTGATGGAGGGGGATAACTACTGGAAACGGTAGCTAATACCGCATAATGTTGAGAAACCAAAGCATGGGACCTTCGGGCCATGCGCCATTTGATGAACCCATATGGGATTAGCTAGTTGGTAGGGTAAAGGCTTACCAAGGCGACGATCTCTAGCTGGTCTGAGAGGATGACCAGCCACACTGGAACTGAGACACGGTCCAGACTCCTACGGGAGGCTGC

>Cluster6040 [Species group=Enterobacteriaceae] [Pyrotag=GPEDTIY04I6KLM]

AGAGTTTGATCCTGGCTCAGATTGAACGCTGGCGGCAGGCCTAACACATGCAAGTCGAACGGTAACAGGAAGCAGCTTGCTGCTTTGCTGACGAGTGGCGGACGGGTGAGTAATGTCTGGGAAACTGCCTGATGGAGGGGGATAACTACTGGAAACGGTAGCTAATACCGCATAACGTCGCAAGACCAAAGAGGGGGACCTTCGGGCCTCTTGCCATCGGATGTGCCCAGATGGGATTAGCTAGTAGGTGGGGTAACGGCTCACCTAGGCGACGATCTCTAGCTGGTCTGAGAGGATGACCAGCCACACTGGAACTGAGACACGGTCCAGACTCCTACGGGAGGCAGC

>Cluster6128 [Species group=Enterobacteriaceae] [Pyrotag=GPEDTIY04ITFFT]

AGAGTTTGATCATGGCTCAGATTGAACGCTGGCGGCAGGCTTAACACATGCAAGTCGAGCGGTAGCACAGGGGAGCTTGCTCCCCGGGTGACGAGCGGCGGACGGGTGAGTAATGTCTGGGAAACTGCCTGATGGAGGGGGATAACTACTGGAAACGGTAGCTAATACCGCATAACGTCGCAAGACCAAAGAGGGGGACCTTCGGGCCTCTTGCCATCAGATGTGCCCAGATGGGATTATCTAGTAGGTGGGGGTAATGGCTCACCTAGGCGACGATCCCTAGCTGGTCTGAGAGGATGACCAGCCACACTGGAACTGAGACACGGTCCAGACTCCTACGGGAGGCAGC

>Cluster7196 [Species group=Enterobacteriaceae] [Pyrotag=GPEDTIY04IXC5O]

AGAGTTTGATCCTGGCTCAGATTGAACGCTGGCGGCATGCTTTACACATGCAAGTCGAACGGCAGCACGGAGAGCTTGCTCCTGGGTGACGAGCGGCGGACGGGTGAGTAATGTCTGGGAAACTGCCTGATGGAGGGGGATAACTACTGGAAACGGTAGCTAATACCGCATAACGTCGCAAGACCAAAGAGGGGGACCTTCGGGCCTCTTGCCATCAGATGTGCCCAGATGGGATTAGCTAGTAGGTGGGGTAACGGCTCACCTAGGCGACGATCCCTAGCTGGTCTGAGAGGATGACCAGCCACACTGGAACTGAGACACGGTCCAGACTCCTACGGGAGGCAGC

>Cluster1-alpha2 [Species group=Alpha-1] [Pyrotag=GPEDTIY04JF6ZL]

AGAGTTTGATCATGGCTCAGAGCGAACGCTGGCGGCATGCTTAACACATGCAAGTCGCACGGACATTTCGGTGTTAGTGGCGGACGGGTGAGTAACGCGTAGGGATCTGTCCATAAGAGGGGGATAACTTTGGGAAACTGGAGCTAATACCGCATGATACCTGAGGGTTAAAGGAGTGATCCGCTTATGGAGGAACCTGCGTTTGATTAGCTAGTTGGTGGGGTAATGGCCTACCAAGGCGATGATCGATAGCTGGTTTGAGAGGATGATCAGCCACACTGGGACTGAGACACGGCCCAGACTCCTACGGGAGGCTGCCGTGACGGCGGCGTCGACGAGGCNCGATAG
