# Supplementary material for: Independent Studies Using Deep Sequencing Resolve the Same Set of Core Bacterial Species Dominating Gut Communities of Honey Bees
Source: PLoS One. 2012 Jul 19;7(7):e41250. doi: 10.1371/journal.pone.0041250 (PMC3400611; doi:10.1371/journal.pone.0041250)
Supplement: Text S2 — Summary of clustering and diversity analyses using alternative quality filter and clustering criteria. (PDF) [file pone.0041250.s003.pdf]

Text S2. Summary of clustering and diversity analyses using alternative quality filter and clustering criteria.

An alternative analysis of the gut-derived pyrotag data was performed to determine whether filtering and clustering methods affect conclusions regarding frequency of reads from known bee bacteria and effects of queen mating on diversity. We required quality score >27 for >97% of a read and read length between 300 and 400 bp. Under these criteria, 138,446 of 217,541 reads were retained. Using Mothur (Schloss et al., 2009) to cluster reads into OTUs at 97% identity yielded1973 OTUs of which 1316 (66.7%) contained only one read. Excluding rare OTUs, by requiring at least 2 sequences in each of at least 2 samples, resulted in retention of 166 OTUs. Excluded OTUs totaled 2.1% of total reads.

These OTUs were assigned to species using methods described in the main text. Overall, 95.0% of the reads corresponded to species known from previous studies of bee guts (versus 94.8% for the analysis described in Results). Of the remaining 5% of reads, 91% were assigned to various Enterobacteriaceae.

The retained 166 OTUs were used in diversity analyses as described for the main analysis of 42 OTUs. In the MRPP analyses, communities in colonies with singly and multiply mated queens are not significantly different ((without outliers excluded, MRPP: T=0.391, A=-0.00457, P=0.58; with outliers excluded MRPP: T=0.626, A=-0.00747, P=0.70). In the NMS analysis, the two colonies types again are not different (Fig. S1; 3D solution, 500 iterations, final stress=8.888, final instability= 0.00165, Monte Carlo P= 0.012. Variation explained: Axis 1 = 22.7%; Axis 2 = 29.2%; and Axis 3 = 40.5%; outliers removed were S5, S7, M10). Likewise, the OTU accumulation curves do not support a difference: confidence interval for the multiply mated queen colonies encompasses the accumulation curve for the singly mated queen colonies. Community richness, measured as Chao 2, is also similar for the two types of colonies: multiply mated group: 147.4 (136.8-174.1, 95% CI); singly mated group 158.5 (144.2-191.4, 95% CI).

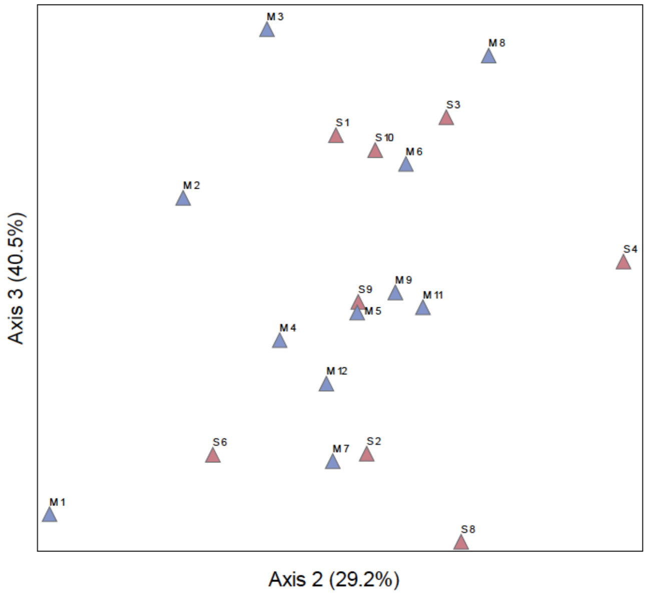

Figure S1. MRPP analysis of bacterial communities from honey bee guts showing distribution across the two major axes of variation. Red and blue symbols represent communities from singly and multiply mated colonies respectively.

Schloss, P.D., et al., (2009) Introducing Mothur: Open-source, platform-independent, community-supported software for describing and comparing microbial communities. Applied and Environmental Microbiology 75:7537-7541.
